# Application of short-term inhalation studies to assess the inhalation toxicity of nanomaterials

**DOI:** 10.1186/1743-8977-11-16

**Published:** 2014-04-04

**Authors:** Robert Landsiedel, Lan Ma-Hock, Thomas Hofmann, Martin Wiemann, Volker Strauss, Silke Treumann, Wendel Wohlleben, Sibylle Gröters, Karin Wiench, Bennard van Ravenzwaay

**Affiliations:** 1 Experimental Toxicology and Ecology, BASF SE, 67056 Ludwigshafen, Germany; 2 Institute for Biological Emission Evaluation, Münster, Germany; 3 Material Physics, BASF SE, Ludwigshafen, Germany; 4 Product Safety, BASF SE, Ludwigshafen, Germany

**Keywords:** Inhalation, Nanomaterial, TiO_2_, ZnO, SiO_2_, BaSO_4_, ZrO_2_, CeO_2_, Rat, Bronchoalveolar lavage, Cytokine/chemokine, Respiratory tract histopathology

## Abstract

**Background:**

A standard short-term inhalation study (STIS) was applied for hazard assessment of 13 metal oxide nanomaterials and micron-scale zinc oxide.

**Methods:**

Rats were exposed to test material aerosols (ranging from 0.5 to 50 mg/m^3^) for five consecutive days with 14- or 21-day post-exposure observation. Bronchoalveolar lavage fluid (BALF) and histopathological sections of the entire respiratory tract were examined. Pulmonary deposition and clearance and test material translocation into extra-pulmonary organs were assessed.

**Results:**

Inhaled nanomaterials were found in the lung, in alveolar macrophages, and in the draining lymph nodes. Polyacrylate-coated silica was also found in the spleen, and both zinc oxides elicited olfactory epithelium necrosis. None of the other nanomaterials was recorded in extra-pulmonary organs. Eight nanomaterials did not elicit pulmonary effects, and their no observed adverse effect concentrations (NOAECs) were at least 10 mg/m^3^. Five materials (coated nano-TiO_2_, both ZnO, both CeO_2_) evoked concentration-dependent transient pulmonary inflammation. Most effects were at least partially reversible during the post-exposure period.

Based on the NOAECs that were derived from quantitative parameters, with BALF polymorphonuclear (PMN) neutrophil counts and total protein concentration being most sensitive, or from the severity of histopathological findings, the materials were ranked by increasing toxic potency into 3 grades: lower toxic potency: BaSO_4_; SiO_2_.acrylate (by local NOAEC); SiO_2_.PEG; SiO_2_.phosphate; SiO_2_.amino; nano-ZrO_2_; ZrO_2_.TODA; ZrO_2_.acrylate; medium toxic potency: SiO_2_.naked; higher toxic potency: coated nano-TiO_2_; nano-CeO_2_; Al-doped nano-CeO_2_; micron-scale ZnO; coated nano-ZnO (and SiO_2_.acrylate by systemic no observed effect concentration (NOEC)).

**Conclusion:**

The STIS revealed the type of effects of 13 nanomaterials, and micron-scale ZnO, information on their toxic potency, and the location and reversibility of effects. Assessment of lung burden and material translocation provided preliminary biokinetic information. Based upon the study results, the STIS protocol was re-assessed and preliminary suggestions regarding the grouping of nanomaterials for safety assessment were spelled out.

## Background

Nanomaterials offer unique mechanical, chemical, electrical or optical properties that are being exploited for a broad spectrum of applications, including a variety of industrial and consumer products and products and devices in the medical field. However, the increased use of nanomaterials has also raised concerns about undesirable human health effects, especially upon inhalation uptake. Information on possible toxic effects of nanomaterials is essential to ensure occupational and consumer safety. Standard toxicological testing methods, such as the 90-day rodent inhalation study, have been recognized as generally being applicable in meeting this request. However, such studies are considerably time-consuming and cost-intensive and not suitable for screening purposes or for the testing of larger numbers of compounds.

In addressing these drawbacks of long-term studies, a rat short-term inhalation study protocol (STIS) was developed within the project NanoCare (http://www.ptj.de/nanocare; of note: all websites were accessed in March 2014) supported by the German Federal Ministry of Education and Research and the European Sixth Framework Programme project NanoSafe2 (http://www.nanosafe.org). The STIS protocol takes into account the specific properties of nanomaterials and encompasses a 5-day inhalation exposure period with a subsequent 3-week post-exposure period. During a first validation of this protocol, nano-TiO_2_ was applied as a model substance [1], and its effects were compared to micron-scale TiO_2_ and micron-scale quartz. The results of this initial study with TiO_2_ stood in good agreement with those obtained in a subchronic 90-day study [2].

The study at hand presents STIS data for nanomaterials, which were tested either during product development, i.e. TiO_2_ (T-lite SF™) and ZnO (NM-111), or in the context of research projects, i.e. nano-CeO_2_, Al-doped nano-CeO_2_, ZrO_2_ (with or without surface modifications), naked amorphous silica and four types of surface-coated silica, and BaSO_4_ (NM-220). Of note, NM-x numberings refer to the respective numberings of OECD reference nanomaterials, as they have been coded in the list of the OECD WPMN Sponsorship Program for the Testing of Manufactured Nanomaterials (http://www.oecd.org/science/nanosafety/ and http://ihcp.jrc.ec.europa.eu/our_activities/nanotechnology/nanomaterials-repository).

The STIS for both CeO_2_ nanomaterials and the ZrO_2_ without surface modification were performed in the context of the above-mentioned NanoCare project, and the STIS for the surface-functionalized ZrO_2_, all SiO_2_ materials and BaSo_4_ were conducted as a part of the ‘nanoGEM’ project (*Nanostrukturierte Materialien – Gesundheit, Exposition und Materialeigenschaften* – ‘Nanostructured Materials – Health, Exposure and Material Characteristics’; http://www.nanogem.de) funded by the German Federal Ministry of Education and Research. For a few of the test materials of the present study, data from chronic and subchronic studies are already available, and a comparison of the results from short-term versus long-term studies has recently been published [3], revealing consistent results regardless of the differing exposure periods.

Coated nano-TiO_2_ is being used as a sunscreen in cosmetic products. Nanosized TiO_2_ has been observed to elicit inflammatory effects [4-6] and morphological changes in the lung [2,7].

Just as TiO_2_, ZnO nanomaterials are used as UV absorbers. In workers, ‘metal fume fever’ effects caused by ZnO have been observed to be potent, but transient; *in vitro,* ZnO nanomaterials have been recorded to be highly toxic to many types of cells with concordant effects observed *in vivo* upon instillation, to the lung tissue [8]. Already at a bolus concentration of 0.3 mg instilled into the rat lung, ZnO nanoparticles induced collagen formation 4 weeks post-instillation [9]. This lung burden can be achieved by 4-week inhalation exposure to 2 mg/m^3^ (6 h/day, 5 days/week), assuming 10% deposition and the clearance rate of a poorly soluble particle. ZnO particles selected for the present study were a coated nano-ZnO (OECD NM-111) and a micron-scale, uncoated ZnO.

Synthetic naked amorphous silica can be subdivided into wet process-manufactured (precipitated) silica and pyrogenic silica. The surface of silica particles can additionally be coated with a variety of organic materials (overview in [10]). Synthetic amorphous silica particles are being used as filling materials in plastics, lacquers, paints and tyres but also in pharmaceuticals, cosmetics, and foods. In contrast to crystalline silica, the biological effects of synthetic naked amorphous silicas appear to be transient and, depending on the concentration, reversible [10-12]. Precipitated uncoated amorphous silica (in the following: SiO_2_.naked) and four surface modifications thereof (SiO_2_.polyacrylate, SiO_2_.polyethyleneglycol (SiO_2_.PEG), SiO_2_.phosphate, SiO_2_.amino) were tested in the present study.

Nanosized BaSO_4_ is being used as a filling material in polymer compositions to increase scratch resistance while conserving transparency. Upon inhalation, micron-scale BaSO_4_ only induces very slight transient inflammatory reactions [13], and it has been used for a long time as a radio-contrast agent for medical diagnostic purposes. By contrast, nanosized BaSO_4_ particles have a much larger surface area and have not yet been tested via the inhalation route.

Industrial areas of use of ZrO_2_ particles include applications in foundry sands, refractories, and ceramics. The material is also used for the coating of hip joint endoprostheses or dental prostheses. ZrO_2_ particles increase durability and scratch resistance of enamels and varnishes. Micron-scale ZrO_2_, as investigated by Pott and Roller [14], possesses a comparatively low carcinogenic potential under severe overload conditions achieved by intratracheal instillation of bolus doses of 60 mg/rat lung. So far, there are no data available on the pulmonary effects of nano-ZrO_2_ particles either without or with surface modification. Hence the nano-ZrO_2_ and surface-modified nano-ZrO_2_ (ZrO_2_.trioxadecanoic acid (ZrO_2_.TODA) and ZrO_2_.polyacrylate) evaluated in the present study provide a first insight on their pulmonary effects.

Nanoparticular CeO_2_ is widely used e.g. in solar cells, as additive in diesel and automotive catalytic converters, as UV absorbent, for glass or ceramic applications and as polishing agent for silicon-wafers. The oxidative and lung toxicity potential of nano-CeO_2_ has recently been demonstrated in a first acute inhalation study [15] in which 641 mg/m^3^ were applied to rats for 4 h in accordance with OECD test guideline (TG) 403. Under these experimental conditions, acute signs of inflammation and oxidative damage including reduced tissue glutathione and increased levels of malondialdehyde were observed. All of these effects were partially reversible within 14 days, whereas the CeO_2_ particles persisted inside the alveoli and lung tissue for a longer period. The results from this first inhalation study were consistent with previous instillation studies [16,17] and with earlier reports on the occurrence of pneumoconiosis in workers exposed to (micron-scale) CeO_2_ particles [18,19]. Two types of CeO_2_ nanomaterials were tested in the present study, which partly differed in chemical composition, with the one being pure and the other being doped with a low percentage of aluminium.

As a rule, the tested aerosol concentration ranges encompass the nuisance dust limit laid down by the German Federal Ministry of Labour and Social Affairs, i.e. 1.25 mg/m^3^ for granular respirable dusts that can reach the alveoli [20,21]. The highest concentrations were selected to challenge the lung clearance mechanism under conditions of beginning lung overload, which was usually at 10 mg/m^3^. For coated nano-TiO_2_ (T-Lite SF™), this concentration had caused adverse effects in previously performed subchronic studies. Lower or higher concentrations were selected, as appropriate, based on existing data from previous toxicological tests, e.g. 50 mg/m^3^ for BaSO_4_. Aerosols were characterized with respect to size distribution and number concentration using ISO-standardized methods or, alternatively, characterization methods recommended by the OECD.

The biological effects of the nanomaterials in rats were determined by analysing the bronchoalveolar lavage fluid (BALF) and blood samples as well as by performing histopathological examinations of all respiratory tract tissues. For all test substances, the organ burden, i.e. the content of test material, was determined in the lung and lung-associated mediastinal lymph nodes, and for most test substances, also in extra-pulmonary organs. Additional information, i.e. cell proliferation rates, an early indicator for epithelial hypertrophy, and apoptotic reactions in the terminal bronchi and alveoli, cytokine profiles in the blood, BALF and/or lung tissue homogenates, or transmission electron microscopic (TEM) evaluation of organs, were additionally collected for selected substances.

In assessing whether effects were to be considered treatment-related or incidental, they were not only compared to the corresponding effects occurring in the control groups, but also to historical control data (*c.f.* Supplementary Information (SI), Additional file 1: Table S1, for historical control ranges of BALF and lung tissue homogenate parameters of male Crl:Wi(Han) rats, 8–12 weeks of age).

## Results

### Characterization of the test materials

**Figure 1:**
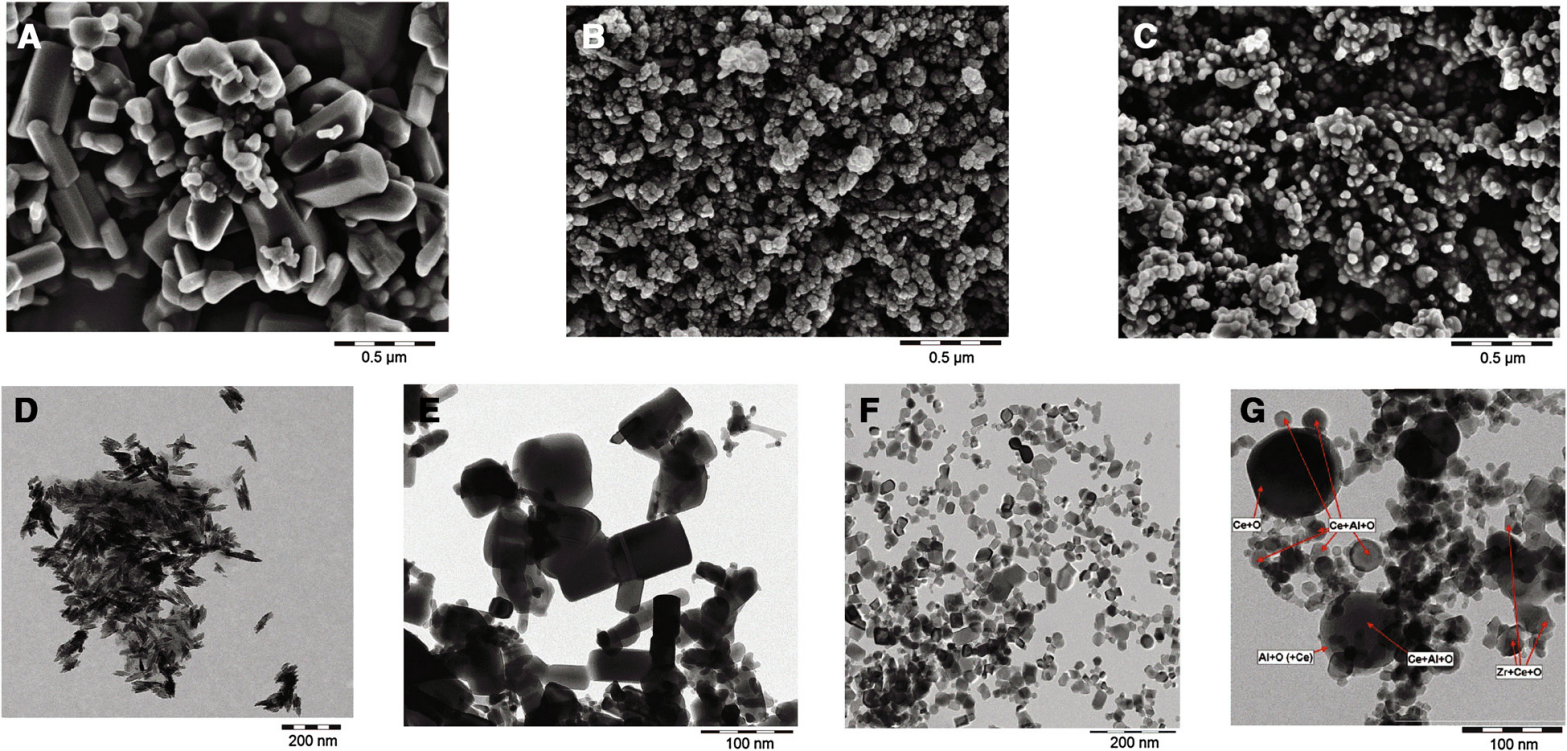
**Scanning and transmission electron microscopy (SEM and TEM) images of the powder test materials. A**: micron-scale ZnO, **B**: BaSO_4_, **C**: nano-ZrO_2_, **D**: coated nano-TiO_2_, **E**: coated nano-ZnO, **F**: nano-CeO_2_, **G**: Al-doped nano-CeO_2_. **A-C**: SEM images. **D-G**: TEM images. Note slightly different sets of scales in order to point to the individual characteristics of the respective test materials.

**Figure 2:**
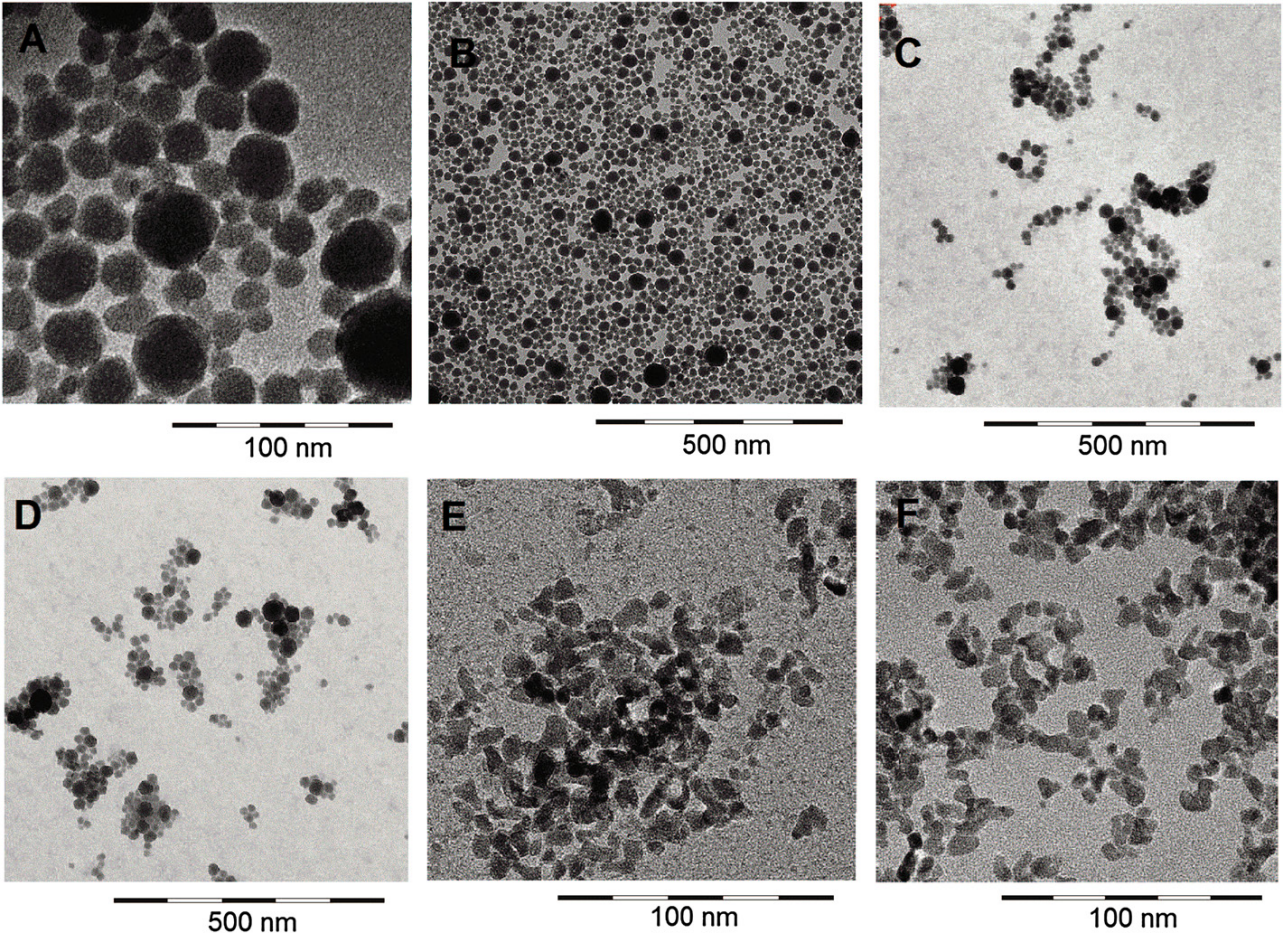
**Scanning electron microscopy images of the suspension test materials. A**: SiO_2_.naked, **B**: SiO_2_.PEG, **C**: SiO_2_.amino, **D**: SiO_2_.phosphate, **E**: ZrO_2_.TODA, **F**: ZrO_2_.acrylate. Note slightly different sets of scales in order to point to the individual characteristics of the respective test materials.

A comprehensive overview of the physico-chemical characteristics of the test materials is provided in Table 1. Details of this characterization have previously been published for those test materials that were delivered as powders (i.e. TiO_2_, both ZnO, both CeO_2_, BaSO_4_, nano-ZrO_2_) [22] and for those materials that were delivered as suspensions (i.e. all SiO_2_; ZrO_2_.TODA; ZrO_2_.acrylate) [23]. Morphological images of all test substances, except for SiO_2_.acrylate, obtained by scanning electron microscopy (SEM) and TEM, are presented in Figures 1 and 2.

**Table 1:** Physico-chemical characterization of the 14 test materials (i.e. 13 nanomaterials and micron-scale ZnO)

	**Al-doped CeO**_**2**_	**CeO**_**2**_	**Uncoated ZrO**_**2**_	**Nano-BaSO**_**4 **_**NM-220**	**TiO**_**2 **_**(T-Lite SF™)**	**coated nano-ZnO NM-111**	**micron-scale ZnO**	**SiO**_**2**_**. naked**	**SiO**_**2**_**. acrylate**	**SiO**_**2**_**. PEG**	**SiO**_**2**_**. amino**	**SiO**_**2**_**. phosphate**	**ZrO**_**2**_**. acrylate**	**ZrO**_**2**_**. TODA**
**PPS/shape** (TEM; nm)	2-160 globular	0-200 globular	25-60 globular	25 globular	15 × 50 Aspect ratio >3, fiber	20-200 mostly globular	50 – 500 globular	15	20	15	15	15	9	9
**Agglomerate size/shape** (SEM; nm)	>20,000	>10,000	1,000 - 5,000	2,800 - 15,000; spheres		>20,000	2,500							
**Crystallite size** (XRD; nm)	23	36	45	36	24	61	>100	n.a.	n.a.	n.a.	n.a.	n.a.	n.a.	n.a.
**Crystalline phase** (XRD)	Cerianite cubic	Cerianite cubic	ZrO2 tetragonal	Barite orthorhombic	Rutile, (minimally anatase)	Zincite, ZnO hexagonal	Zincite, ZnO hexagonal	n.a.	n.a.	n.a.	n.a.	n.a.	n.a.	n.a.
**Pore sizes** (Hg porosimetry; nm)	30; 800; 40,000	30; 20,000	40; 1,000; 15,000	30; 5,000	20; 30,000	30; 40,000	5; 200; 7,000	n.a.	n.a.	n.a.	n.a.	n.a.	n.a.	n.a.
**Specific surface area** (BET nitrogen adsorption and Hg intrusion porosimetry; m^2^/g)	46.0 (62)	33.0 (34)	24.9 (29)	41.4 (33)	100.0 (82)	12.0 (20)	5.6 (55)	n.a.	n.a.	n.a.	n.a.	n.a.	n.a.	n.a.
**Surface chemistry** (XPS; supported by SIMS; as necessary; Atom%/qualitative)	Ce: 21; Al: 9; O: 56; C: 9; Zr: 4; N: 1	Ce: 16; O: 61; C: 9 (C-C, O-C = O); Al: 9; Zr: 5	Zr: 24; O: 53; C: 19 (C-C, C-O, O-C = O); N: 3; Al: 1	Ba: 13; O; 52; C: 17; S: 11; Cl: 3; P: 3; N: 1	Ti: 16; O: 63; C: 9; Al: 7; Si: 5; Na: <1; dimethicone/methicone copolymer as surface coating	Zn: 1; C: 64; (C-C, C-O, peptide) O: 19; N: 12; Na: 2; P: 2; Cl: 1; 3.5% triethoxy-octylsilane	n/d	Si: 29; O: 66; C: 4 (C-C, C-H, C-O, C = O) Na: 1	Si: 21; O: 54C: 24 (C-C, some C-O, C = O); Na: 1; SIMS: poly-methacrylic acid/3-methac-ryloxypropyl	SIMS: expected fragments of PEG500 (CH_2_CH_2_O)	SIMS: expected fragments of aminosilane	Si: 29; O: 66; C: 4.6; Na: 0.5; expected P, N not detec-ted by XPS, but PO_2_, PO_3_ fragments by SIMS	Zr 23; O: 58; C: 19; SIMS: expected acrylic acid	Zr; 24; O: 63; C: 11; N: 0.7; S: 0.2; SIMS: expected trioxa-decanoic acid
**Photocatalysis: photon efficiency,** unitless^a^	8.8E-04	1.6E-03	7.0E-04	1.1E-03	5.3E-04	n.m. (hydrophobic)	5.0E-01	1.7E-5	5.9E-5	6.2E-5	8.8E-5	1.9E-4	2.9E-5	5.8E-5
**Surface charge** Iso-electric point/Mobility at pH 7	8.5/1.4 (μm/s)/V/cm)	7.5/0.5 (μm/s)/V/cm)	6.5/-0.9 (μm/s)/V/cm)	3.3/-2.2 (μm/s)/V/cm)	6.5/-0.2 (μm/s)/V/cm)	n.m. (agglo-merated)	n.m. (agglo-merated)	<1/-	<1/-	4/-	7.2/-	<1/-	<1/-	7.1/-
**Zeta-potential at pH 7 (mV)**	18	6	−12	−28	−3			−38	−47	−26	0	−43	−39	−6
**Surface reactivity** (ESR + CPH)^b^								4 (p-f s: 0.8)	n.d.	1 (p-f s: 3.4)	1 (p-f s: 1.1)	2.2 (p-f s: 1.2)	1 (p-f s: 2)	0.54 (p-f s: 5.7)
**Formation of OH radicals (ROS)** ESR + DMPO^b^								11 (p-f s: 6.3)	n.d.	11 (p-f s: 13)	21 (p-f s: 5.2)	19 (p-f s: 5)	3.6 (p-f s: 1.5)	0.94 (p-f s: 1.3)
**Particle size/dispersability in water**	603/20	90/3.0	1,500/38; <0.1 wt% <100 nm	116/4.6	33/1.7	Agglom.; <0.01 wt% <100 nm	5,100 /20; <0.1 wt% <100 nm	19/1	23/1	21/1	20/1	20/1	27/3	11/1
**in DMEM/FCS** (AUC; D_50_ (nm)/AAN (qualitative))	94/3.1	54/1.8	87/2.2	285/11	3,900/195; 2 wt% <100 nm	550/18	950/3.8	420/28	26/1	3200/213	1350/90	30/2	315/32	860/86
**Solubility** in water and in DMEM/FCS	Ce, Al <0.1 ppm	Ce <0.1 ppm	Zr <0.1 ppm	Ba 6 ppm	Ti 5 ppm Ti 5 ppm	Soluble at pH < 6	Zn <5 ppm Zn 50 ppm							

^a^Catalytic events per photon impinging on the surface, as specified in DIN 52980.

^b^Surface reactivity and ROS formation were determined relative to the ‘reference material’ deuterium oxide (D_2_O; ^2^H_2_O). Assuming a 30% variability of the methodology, only measurements >1.3 are considered relevant. Of note, this value should only serve as a guiding principle, and not as an absolute value.

*Abbreviations*: *AAN* average agglomeration number; derived from the ratio of the volume-based median particle size to the average equivalent spherical volume derived from the BET gas adsorption, *AUC* analytical ultracentrifugation, *BET* method of Brunauer-Emmett-Teller, D_50_ medium value of the particle size distribution, *DMEM/FCS* Dulbecco’s Modified Eagle Medium supplemented with 10% fetal calf serum, *ESR + CPH* Electron spin resonance making use of centrophenoxine spin traps, *ESR + DMPO* Electron spin resonance making use of dimethyl-pyrroline-N-oxide spin traps, *n.a.* not applicable, *n.d.* not determined, *n.m.* not measurable, *p-f s* particle-free supernatant, *PPS* primary particle size, *ROS* reactive oxygen species, *SEM* scanning electron microscopy, *SIMS* secondary ion mass spectrometry, *TEM* transmission electron microscopy, *XPS* x-ray photoelectron spectroscopy, *XRD* x-ray diffusion.

### Characterization of the test aerosols

The achieved aerosol concentrations were generally close to the corresponding target concentrations (Table 2). Evaluation of the particle size distribution confirmed that the aerosols were respirable for rats. Size measurements in the submicrometer range conducted with the Scanning Mobility Particle Sizer (SMPS) revealed that the aerosols consisted of few primary particles in the range of 14 to 90 nm and of agglomerates with diameters up to 3 micrometres, with the majority of the agglomerates (typically 80%) being in the sub-micrometre range.

**Table 2:** Targeted and measured test substance concentrations and particle size distributions

**Test substance**	**Targeted concentrations (mg/m**^**3**^**)**	**Measured concentrations, mean ± SD (mg/m**^**3**^**)**	**MMAD (μm)/GSD**		**Particle count concentration measured by SMPS (particle/cm**^**3**^**)**	**Particle count Median (μm)**
			**Measurement 1**	**Measurement 2**		
**Coated nano-TiO**_**2**_	0.5	0.6 ± 0.1	0.8/3.4	0.7/4.4	33905	0.131
	2	2.0 ± 0.1	0.4/3.1	0.2/4.0	114889	0.153
	10	10.7 ± 1.2	0.4/3.0	0.4/3.6	205660	0.167
**Micron-scale ZnO**	12.5	15.3 ± 1.6	1.0/2.2	1.1/2.3	219031	0.167
**Coated nano-ZnO (NM-111)**	0.5	0.6 ± 0.2	0.9/2.4	1.3/2.4	22126	0.144
	2.5	2.8 ± 0.6	0.8/2.5	0.8/2.8	87044	0.177
	12.5	13.8 ± 2.0	0.8/2.4	0.9/2.4	159381	0.198
**SiO**_**2**_**.naked**	0.5	0.5 ± 0.1	1.3/3.0	1.7/3.4	20167	0.106
	2.5	2.4 ± 0.1	1.0/2.3	1.2/2.2	47866	0.101
	10	10.4 ± 1.3	1.3/2.2	1.5/2.3	130972	0.127
	50	52.6 ± 4.3	2.0/2.8	2.2/2.6	172204	0.114
**SiO**_**2**_**.acrylate**	0.5	0.6 ± 0.1	1.1/ 2.4	1.2/2.3	13607	0.090
	2.0	2.1 ± 0.2	1.0/2.3	1.0/2.3	30623	0.100
	10	9.7 ± 0.4	1.1/2.8	1.1/2.3	159381	0.110
**SiO**_**2**_**.PEG**	2	2.05 ± 0.12	1.0/2.8	1.0/2.5	17080	0.093
	10	10.0 ± 1.4	1.0/2.7	1.1/2.7	73384	0.100
	50	54.1 ± 1.0	1.3/2.8	1.3/2.8	164639	0.125
**SiO**_**2**_**.phosphate**	2	2.9 ± 0.6	0.8/3.6	0.9/2.6	109566	0.09
	10	10.0 ± 1.2	1.4/2.6	1.3/2.3	105863	0.09
	50	51.5 ± 5.4	1.4/2.5	1.6/2.4	308408	0.10
**SiO**_**2**_**.amino**	2	2.1 ± 0.4	0.8 /2.4	0.9/4.0	11036	0.09
	10	10.2 ± 1.4	1.8/2.0	1.4/2.2	146680	0.10
	50	50.4 ± 3.7	1.7/2.7	1.3/2.8	282401	0.10
**Nano-BaSO**_**4 **_**(NM-220)**	2	2.5 ± 0.3	1.3/2.4	1.2/2.2	71945	0.173
	10	13.1 ± 0.7	1.5/2.1	1.4/2.3	245438	0.198
	50	53.4 ± 9.7	1.1/2.2	0.9/2.3	258642	0.188
**Nano-ZrO**_**2**_	0.5	0.5 ± 0.1	1.6/2.1	1.3/2.0	8580	0.091
	2.5	2.6 ± 0.3	1.3/2.0	1.4/2.3	16024	0.138
	10	9.6 ± 1.2	1.8/2.2	2.0/1.8	76924	0.149
**ZrO**_**2**_**.TODA**	2	2.0 ± 0.1	1.3/4.0	1.2/3.9	59496	0.08
	10	10.6 ± 0.3	1.0/4.7	1.1/4.2	72398	0.11
	50	52.2 ± 1.1	1.5/3.3	1.2/4.3	124267	0.13
**ZrO**_**2**_**.acrylate**	2	1.9 ± 0.1	0.6 /2.9	0.7/2.7	54218	0.06
	10	10.1 ± 1.0	1.0/2.6	0.8/2.8	133269	0.06
	50	50.5 ± 4.7	1.4/2.7	1.3/3.4	166557	0.06
**Nano-CeO**_**2**_	0.5	0.8 ± 0.3	0.6/2.4	0.7/2.9	47745	0.111
	2.5	3.0 ± 0.2	0.9/2.3	0.8/2.3	126354	0.144
	10	11.6 ± 0.5	0.8/2.5	0.7/2.4	458415	0.172
**Al-doped nano-CeO**_**2**_	0.5	0.6 ± 0.3	1.3/2.1	1.1/2.3	39695	0.252
	2	2.1 ± 0.5	2.2/1.9	1.8/1.9	-	-
	10	9.2 ± 2.6	2.4/2.1	1.8/1.9	82383	0.200

### Toxicological effects

**Figure 3:**
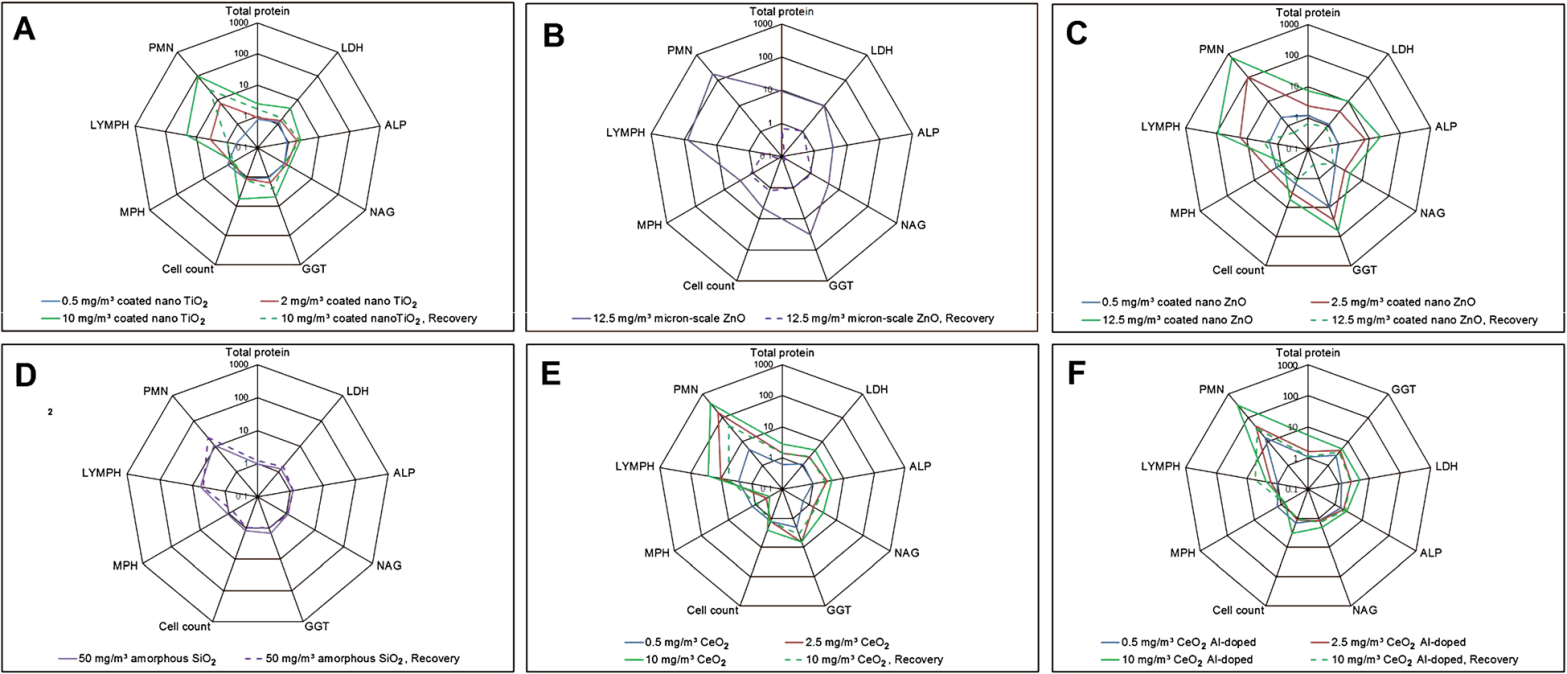
**Comparison of changes in BALF. A**: coated nano-TiO_2_. B: micron-scale ZnO; **C**: coated nano-ZnO; **D**: SiO_2_.naked; **E**: nano-CeO_2_; **F**: Al-doped nano-CeO_2_. Changes are shown as x-fold differences compared to the corresponding control values using a logarithmic scaling.

An overview of the toxicological effects elicited by the 13 nanomaterials and micron-scale ZnO is provided in Table 3, with details on affected BALF parameters presented in Figures 3A-3F and the Supplementary Information (SI) Additional file 1: Tables S1-S9. The incidences and severities of the histological findings induced by the TiO_2_, ZnO, and CeO_2_ test materials are summarized in SI, Additional file 2: Tables S10-S15. The following sub-sections present the toxicological effects observed for each test material, and the subsequent Organ burden analysis summarizes the outcome of the organ burden analysis (Table 4). An overview of the study design, indicating which specific examinations were conducted in the animal groups treated with either of the test materials, is provided in Table 5.

**Table 3:** Summary of the test results obtained for 13 nanomaterials and micron-scale ZnO in rat short-term inhalation studies

**Test material**	**Target con-centrations [mg/m**^**3**^**]**	**NOAEC (mg/m**^**3**^**)**	**Findings in the BALF**	**Pathological and histological findings**	**Reversibility of effects**
Coated nano-TiO_2_ (T-Lite SF™)	0.5, 2.0, 10.0	0.5	Increased total cell counts, and PMN neutrophils, monocytes, total protein, GGT, LDH, ALP and NAG (cytokines not measured)	Lung: pigment-loaded alveolar macrophages and slight diffuse histiocytosis	Slight increases in BALF parameters remaining
Micron-scale ZnO	12.5	n.a.	Increased total cell counts and PMN neutrophils, lymphocytes, monocytes, total protein, GGT, LDH, ALP and NAG. Many mediators increased; above 10-fold as compared to respective control values: clusterin; CRP; MCP-1; MCP-3; MDC; MPO; OPN. (Monocyte data not shown in Additional file 1: Table S1)	Nasal cavity: severe multifocal necrosis of olfactory epithelia Lung: Increased absolute (+27%) and relative lung (+34%) weight, bronchoalveolar hyperplasia, histiocytosis, granulocytic infiltration Mediastinal lymph nodes: lympho-reticulo-cellular hyperplasia	Slight to moderate histiocytosis in the lung and irregularities of olfactory epithelium remaining
Coated nano- ZnO (NM-111)	0.5, 2.5, 12.5	0.5	Increased total cell counts and PMN neutrophils, lymphocyte, monocyte, total protein, GGT, LDH, ALP and NAG. Many mediators increased; above 10-fold at highest concentration as compared to respective control values: CINC-1; clusterin; cystatin C; GCP-2; MCP-1; M-CSF; MDC; MPO; OPN	Nasal cavity: moderate multifocal necrosis of olfactory epithelia Lung: histiocytosis, granulocytic infiltration Mediastinal lymph nodes: lympho-reticulo-cellular hyperplasia	Moderate histiocytosis in the lung and irregularities of olfactory epithelium remaining
SiO_2_.naked	0.5; 2.5; 10.0; 50.0	2.5	Slightly increased PMN neutrophils and lymphocytes	Slightly increased neutrophil counts in blood after the end of exposure (data not shown) Respiratory tract: Multifocal macrophage aggregates; Exacerbation towards a slight multifocal inflammation after 3 weeks (data not shown)	Exacerbation towards a slight multifocal pulmonary inflammation
SiO_2_.acrylate	0.5; 2.0; 10.0	local effects: ≥ 10; systemic effects: 0.5	No adverse findings	Respiratory tract: no adverse effects, Spleen: increased weight (+ 25%) without histological correlate; particles and high numbers of thrombocytes in the spleen, detected by TEM	Full reversibility of splenetic effects; no pulmonary effects at any time point
SiO_2_.PEG	2.0; 10.0; 50.0	≥ 50	No adverse findings	No adverse findings	n.a.
SiO_2_.phosphate	2.0; 10.0; 50.0	≥ 50	No adverse findings	No adverse findings	n.a.
SiO_2_.amino	2.0; 10.0; 50.0	≥ 50	No adverse findings	No adverse findings	n.a.
Nano-BaSO_4_ (NM-220)	2.0; 10.0; 50.0	≥ 50	No adverse findings	No adverse findings	n.a.
Nano-ZrO_2_	0.5, 2.5, 10.0	≥ 10	No adverse findings	No adverse findings	n.a.
ZrO_2_.TODA	2.0; 10.0; 50.0	≥ 50	No adverse findings	No adverse findings	n.a.
ZrO_2_.acrylate	2.0; 10.0; 50.0	≥ 50	No adverse findings	No adverse findings	n.a.
Nano-CeO_2_	0.5, 2.5, 10.0	< 0.5	Changes of all cytological and biochemical parameters in BALF; increased levels of Changes of CINC-1, IFNγ, IL-1α, MCP-1, M-CSF, in BALF and lung tissue	Lung: Particles in macrophages (recovery group: additionally mild histiocytosis)	Partial regression of BALF effects; mild diffuse or multifocal alveolar histiocytosis remaining
Al-doped nano- CeO_2_	0.5, 2.0 10.0	< 0.5	Changes of all cytological and biochemical parameters in BALF, increased MCP-1 and CINC-1 in BALF, increased IL1-α in lung tissue	Lung: single or aggregated particle-loaded macrophages	Partial regression of BALF effects; particle-loaded alveolar macrophages remaining

n.a.: not applicable.

**Table 4:** Measured test substance deposition in the lung, expected deposition calculated by the Multiple Path Particle Dosimetry (MPPD) model, measured (absolute and relative) decrease of lung burden (clearance) after the recovery period

**Test substance**	**Assumed 100% deposition (mg)**	**(Lung burden) Measured test material on study day 5 (μg/lung)**	**Measured deposition (%)**	**Calculated deposition (%)**	**Measured lung burden after recovery period (μg/lung)**	**% Decrease of lung burden after recovery period**^**a**^
**Coated nano-TiO**_**2 **_**(T-lite SF™ containing 82% TiO**_**2 **_**and 10% Al(OH)**_**3**_**)**	3.85	459.0 ± 71.3 (TiO_2_) 49.1 ± 7.6 (Al(OH)_3_)	12.4%^b^	19.1%	467.4 ± 43.4 (TiO_2_) 46.3 ± 5.0 (Al(OH)_3_)	+1%
**Micron-scale ZnO**	5.508	82.3 ± 12.5	1.5%	10.3%	27.1 ± 1.5	n.a.
**Coated nano-ZnO (NM-111)**	0.216	33.9 ± 7.0	15.7%	12.4%	25.4 ± 1.3	n.a.
	1.008	123.4 ± 28.4	12.2%	13.5%	26.3 ± 1.5	n.a.
	4.968	428.2 ± 19.4	8.6%	13.4%	28.4 ± 4.1	n.a.
**SiO**_**2**_**.naked**	18.936	342.3 ± 10.7	1.8%	5.1%	208.2 ± 16.1	−39%
**SiO**_**2**_**.acrylate**	0.216	18.6 ± 6.2	8.6%	7.1%	15.7 ± 6.9	−16%
	0.756	37.1 ± 6.9	4.9%	7.9%	30.0 ± 7.7	−19%
	3.492	200.0 ± 30.9	5.7%	6.8%	132.1 ± 43.3	−34%
**SiO**_**2**_**.PEG**	0.738	59.2 ± 11.0	8.0%	8.8%	25.7 ± 2.1	−57%
	3.60	182.6 ± 27.1	5.1%	8.8%	117.0 ± 45.7	−36%
	19.476	834.3 ± 206.3	4.3%	6.5%	370.8 ± 40.5	−56%
**SiO**_**2**_**.phosphate**	1.044	71.3 ± 11.0	6.8%	10.9%	47.8 ± 3.3	−33%
	3.60	194.7 ± 11.9	5.4%	6.1%	104.8 ± 21.4	−46%
	18.54	499.2 ± 30.9	2.7%	6.2%	303.8 ± 91.1	−39%
**SiO**_**2**_**.amino**	0.756	87.0 ± 14.2	11.5%	10.5%	52.1 ± 9.6	−41%
	3.672	295.9 ± 81.7	8.1%	5.3%	154.7 ± 25.7	−48%
	18.144	741.6 ± 157.7	4.1%	5.4%	474.2 ± 155.1	- 36%
**Nano-BaSO**_**4 **_**(NM-220)**^**c**^	19.224	1055.7	5.5%	9.6%	239.7	−77%
**Nano-ZrO**_**2**_	0.18	18.0 ± 5.6	10.0%	7.3%	6.8 ± 3.6	−62%
	0.936	20.3^d^ 117.5	2.2% 12.6%	8.2%	29.3 ± 6.2	−75%
	3.456	270.6 ± 28.9	7.8%	6.7%	157.6 ± 19.0	−42%
**ZrO**_**2**_**.TODA**	0.720	83.3 ± 6.4	11.6%	10.9%	41.9 ± 5.4	−50%
	3.816	233.2 ± 6.9	6.1%	10.9%	87.3 ± 62.8	−63%
	18.792	693.4 ± 121.8	3.7%	7.7%	520.5 ± 82.0	- 25%
**ZrO**_**2**_**.acrylate**	0.684	7^e^	1.0%	16.0%	LOQ	
	3.636	70	1.9%	11.1%	49	- 30%
	18.18	169	0.9%	7.7%	190	+ 12%
**Nano-scale CeO**_**2**_	0.277	52.0 ± 5.0	18.8%	16.6%	44.2 ± 4.4	−15%
	1.069	165.8 ± 18.4	15.5%	14.3%	157.6 ± 18.7	−5%
	4.176	417.6 ± 44.3	10.0%	15.4%	470.9 ± 51.2	+13%
**Al-doped nano-scale CeO**_**2**_^**c**^	3.312	326.8	7.8%	6.8%	304.7	−7%

n.a.: Not applicable: Zn content returned to control levels in all concentration groups. No relative decrease of lung burden was calculated due to high endogenous content of Zn.

LOQ: limit of quantification.

^a^Recovery period for SiO_2_.acrylate was 14 days after the final exposure. For all other substances, the recovery period was 21 days.

^b^Total amount of TiO_2_ and Al(OH)_3_ was assumed as total deposition.

^c^Only one rat was examined.

^d^Only two samples were examined which differed considerably and hence are presented as individual animal data.

^e^Data for one animal, LOQ in the other two.

**Table 5:** Study design

**Test substance**	**Coated nano-TiO**_**2**_	**SiO**_**2**_**.acrylate**	**Micron-scale ZnO; Coated nano-ZnO (NM-111)**	**Nano-CeO**_**2**_**; Nano-ZrO**_**2**_	**SiO**_**2**_**.naked; BaSO**_**4 **_**(NM-220); Al-doped nano-CeO**_**2**_	**SiO**_**2**_**.PEG; SiO**_**2**_**.phosphate; SiO**_**2**_**.amino; ZrO**_**2**_**.TODA; ZrO**_**2**_**.acrylate**
**Study element/part**						
Recovery period	21	14	21	21	21	21
Necropsy and histology of respiratory tract	√^a^	√^b^	√^b^	√^b^	√^c^	√^a^
Histology of brain	n.d.	√^b^	√^b^	√^b^	n.d.	√^a^
Cell proliferation and apoptosis	n.d.	n.d.	√^b^	√^b^	n.d.	n.d.
Electron microscopy	n.d.	√ ^a^	n.d.	n.d.	n.d.	n.d.
BALF cytology, protein and enzyme activities^d^	√	√	√	√	√	√
BALF cytokines and chemokines^d^	n.d.	M-CSF, TGF-β, MIP-2, MCP-1, IL-1α	68 cytokines, chemokines and inflammation-relevant hormones and enzymes^e^	68 cytokines, chemokines and inflammation-relevant hormones and enzymes^e^	Clusterin; MCP-1; CINC-1/IL-8; M-CSF; OPN	MCP-1; CINC-1 /IL-8; M-CSF; OPN
Cytokines and chemokines in lavaged lung tissue^d^	n.d.	n.d.	n.d.	68 cytokines, chemokines and inflammation-relevant hormones and enzymes^e^	IL-1α TNF-α	IL-1α TNF-α
Hematology according to OECD TG 412; c-reactive protein and haptoglobin^d^	n.d.	√	√	√	√	√
Organ burden^a, f^	√	√	√	√	√	√

n.d.: not determined.

^a^For these investigations, altogether 3 animals per concentration and time point were used.

^b^For these investigations, altogether 6 animals per concentration and time point were used.

^c^For this investigations, 3 animals per concentration and time point were used in the BaSO_4_ and Al-doped CeO_2_ test runs, whereas 6 animals per concentration and time point were used in the SiO_2_.naked test run.

^d^For these investigations, altogether 5 animals per concentration and time point were used.

^e^*c.f.* Evaluation of bronchoalveolar lavage fluid (BALF) and lung tissue homogenates.

^f^For all test materials, tissue contents of the respective main element were determined in the lung and lung-associated mediastinal lymph nodes. For coated nano-TiO_2_, BaSO_4_, Al-doped CeO_2_, nano- and micron-scale ZnO, additionally, organ burden of the extra-pulmonary organs was assessed.

*Abbreviations*: *CINC-1/IL-8* cytokine-induced neutrophil chemoattractant-1, the rat homologue to IL-8, *IL* interleukin, *M-CSF* macrophage colony stimulating factor, *MCP*, monocyte chemoattractant protein, *MIP* macrophage inflammatory protein, *OPN* osteopontin, *TNF-α* tumor necrosis factor-α.

#### Coated nano-TiO_2_ (T-Lite SF™)

Inhalation exposure to 10 mg/m^3^ coated nano-TiO_2_ caused markedly increased polymorphonuclear (PMN) neutrophil and monocyte counts in the **BALF** differential cell counts, accompanied by increased total cell counts, enzyme and total protein levels 24 h after the final exposure (i.e. in the exposure groups). Of note, cytokines and chemokines were not assessed for the coated nano-TiO_2_ test groups. Slight increases of a number of BALF parameters were also recorded for the rats of the mid test substance concentration group exposed to 2 mg/m^3^ coated nano-TiO_2_ (Table 3; Figure 3A; and Additional file 1: Table S1, Additional file 1: Table S2). Slightly increased BALF parameters were still observed three weeks post-exposure (i.e. in the recovery groups).

**Histological examination of the lungs** of the high concentration test group (10 mg/m^3^) revealed numerous pigment-loaded alveolar macrophages within the alveoli and slight diffuse histiocytosis, whereas the pulmonary epithelium appeared unchanged. No treatment-related effects were found in the upper airways (i.e. nasal cavity, larynx level, trachea, and carina) or in the mediastinal lymph nodes (Table 3; and Additional file 1: Table S1, Additional file 2: Table S10). Overall, coated nano-TiO_2_ caused mild pulmonary inflammation that was not fully reversible, with a **NOAEC** of 0.5 mg/m^3^. This finding is consistent with previous STIS data evaluating TiO_2_ P25 [1].

#### Micron-scale ZnO and coated nano-ZnO (NM-111)

No clinical signs of toxicity, but markedly decreased **body weight gain**, were observed in the animal groups exposed to 2.5 and 12.5 mg/m^3^ coated nano-ZnO or 12.5 mg/m^3^ micron-scale ZnO 24 h after the final exposure. This effect was assessed as being a result of the systemic toxicity of released Zn^2+^ ions. Body weight gain returned to the control value at the end of the post-exposure period (data not shown).

Hematology parameters, acute phase protein and cytokine levels in the **blood** were not affected in any of the ZnO-treated rats. Increased **absolute and relative lung weights** (by approx. 30%) were observed in animals exposed to micron-scale ZnO (data not shown).

Inhalation exposure to both coated nano-ZnO and micron-scale ZnO caused pulmonary inflammation, which was characterized by considerably increased PMN neutrophil and lymphocyte counts in the differential cell counts, and increased total cell counts in the **BALF**, which were dose-dependent for coated nano-ZnO (Table 3; Figure 3B; and Additional file 1: Table S1, Additional file 1: Table S3) and accompanied by elevated BALF enzyme activities, total protein, and chemokine and cytokine concentrations. Regarding the latter, the levels of 25 and 26, respectively, of a total of 68 examined cell mediators examined (Additional file 1: Table S3) were significantly increased after the final exposure to 12.5 mg/m^3^ coated nano-ZnO or micron-scale ZnO. For coated nano-ZnO, most parameters were also increased at 2.5 mg/m^3^. Even at the lowest aerosol concentration of 0.5 mg/m^3^ coated nano-ZnO, the BALF enzyme glutamyltransferase (GGT), and the inflammatory mediators cytokine-induced neutrophil chemoattractant-1 (CINC-1; the rat homologue to interleukin (IL)-8), clusterin, and tissue inhibitor of metalloproteinases-1 (TIMP-1) were increased. Almost all BALF parameters returned to the respective levels of the control group after the 3-week post-exposure period. Overall, the pattern of changes was similar for both types of ZnO, with stronger effects elicited by coated nano-ZnO (Table 3; and Additional file 1: Table S1, Additional file 1: Table S3).

**Histological examination of the lungs** of the rats exposed to either micron-scale or coated nano-ZnO revealed pronounced PMN neutrophil, macrophage and lymphocyte infiltration (in accordance with the findings in the BALF), and a moderate multifocal rise in the number of alveolar macrophages, accompanied by an activation of the mediastinal lymph nodes. Most effects were fully reversible within the post-exposure period, after which only slight or moderate histiocytosis persisted in all animals. Overall, the histopathological effects caused by both substances were very similar, but compared to micron-scale ZnO, coated nano-ZnO-mediated effects were less severe or had a lower incidence (Table 3; and Additional file 1: Table S1, Additional file 2: Table S11).

In the **upper respiratory tract, i.e. the nasal cavity,** of all rats exposed to the ZnO materials multifocal necrosis of the olfactory epithelium was recorded that was moderate upon exposure to coated nano-ZnO and severe upon exposure to micron-scale ZnO. In test groups treated with 12.5 mg/m^3^ micron-scale ZnO or coated nano-ZnO, slight irregularities of the olfactory epithelium remained visible after the three-week exposure-free period (Table 3; and Additional file 1: Table S1, Additional file 2: Table S12).

In the animals exposed to coated nano-ZnO or micron-scale ZnO increased **cell proliferation in the terminal bronchioli** were observed, which were concentration-dependent for coated nano-ZnO. Micron-scale ZnO also caused increased cell proliferation rates in the **large bronchi and in the alveoli**. In general, a higher proliferation rate was recorded after exposure to 12.5 mg/m^3^ micron-scale ZnO than to the same concentration of coated nano-ZnO (Table 3; and Additional file 1: Table S1, Additional file 2: Table S13). **Apoptosis** was only observed in the test substance groups treated with 12.5 mg/m^3^ of micron-scale ZnO or coated nano-ZnO (data not shown).

Taking into account the changes in the BALF and the morphological findings in the lungs, mediastinal lymph nodes and nasal cavities, the **NOAEC** for coated nano-ZnO was assessed as being 0.5 mg/m^3^. For micron-scale ZnO, no NOAEC was set since it was only tested at one (high) concentration. Based upon the observed results, its NOAEC is likely to be in the same range as the one assessed for coated nano-ZnO, since both materials induced similar effects. While alterations of BALF parameters were more pronounced for coated nano-ZnO than for micron-scale ZnO, coated nano-ZnO elicited less pronounced adverse effects in the upper airways and only minor effects in regard to cell proliferation. These differences can most likely be explained by a reduced dissolution rate due to the coating of the nanoparticles. Similarly, coated nanosized ZnO NM-111 was slightly less toxic in rat precision-cut lung slices than uncoated nanosized ZnO (NM-110) [24].

#### Amorphous silica-based materials

##### Non-coated amorphous silica (SiO_2_.naked)

Inhalation exposure to an aerosol concentration of 50 mg/m^3^ naked amorphous silica caused marginal systemic inflammation, evidenced by slight and transient increases in granulocyte counts in the **blood** (data not shown). Increased PMN neutrophil and lymphocyte counts were present in the **BALF** of this high concentration test group shortly after exposure and (in the 10 and 50 mg/m^3^ test groups) 3 weeks post-exposure. **Histologically**, multifocal macrophage aggregates were observed in the lung shortly after exposure. This finding exacerbated towards a slight multifocal pulmonary inflammation by the end of the 3-week exposure free period (Table 3).

At concentrations up to 50 mg/m^3^, SiO_2_.naked did not induce any significant changes of **BALF** cytokines or chemokines in the exposure groups, i.e. those rats that were euthanized shortly after the final exposure, or in the recovery groups that were euthanized after the 3-week post-exposure period (Additional file 1: Table S12). The **NOAEC** of SiO_2_.naked was assessed as being 2.5 mg/m^3^.

By comparison, Arts et al. [12] submitted three different synthetic amorphous silicas and quartz to a STIS with 3-month post-exposure period. For all three amorphous silica, effects were observed at concentrations that were significantly lower than those from previously published studies investigating comparable substances or from the STIS performed within the present study: While only mild effects were observed in the BALF after exposure to 10 or 50 mg/m^3^ naked amorphous silica in the present study, marked effects were already detected after exposure to 25 mg/m^3^ of all three silicas investigated by Arts and co-workers [12]. These differences in toxicity can most likely be explained by differences in material properties.

##### Polyacrylate-coated amorphous silica (SiO_2_.acrylate)

Inhalation exposure to aerosol concentrations of up to 10 mg/m^3^ polyacrylate-coated amorphous silica did not lead to any biologically relevant changes of **BALF** parameters at any time point (Table 3; and Additional file 1: Table S1, Additional file 1: Table S4), nor were there any changes in hematology parameters or in the acute phase proteins in the **blood**. However, absolute **spleen weights** were increased by 37% and 30% in animals exposed to 2 and 10 mg/m^3^ SiO_2_.acrylate, respectively, and their relative spleen weights were increased by 35% and 26%, respectively. By contrast, the absolute and relative spleen weights of the recovery groups were comparable to the corresponding control values. The increased spleen weights of the exposure groups were assessed as compound-related since the inter-individual variation was small. No morphological changes were detected in the lung upon **histopathological evaluation**.

**Figure 4:**
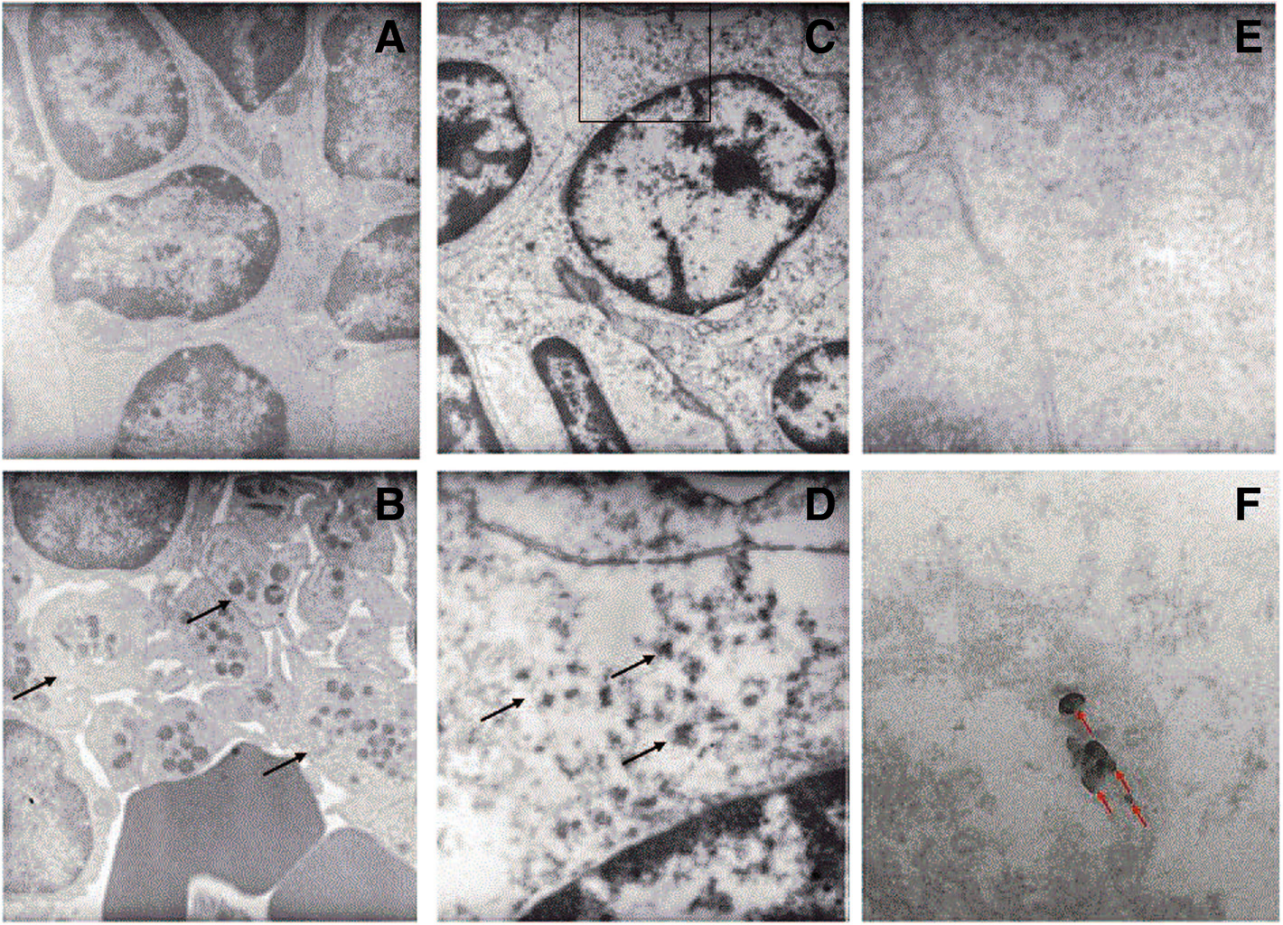
**Transmission electron microscopy of the spleen. A**: control animal, magnification 8000×; **B**: animal exposed to 10 mg/m^3^ SiO_2_.acrylate, magnification 8000×. Image showing accumulation of large numbers of thrombocytes (arrows); **C**: animal exposed to 10 mg/m^3^ SiO_2_.acrylate, magnification 8000×. The lymphocytic cytoplasm is less homogenous than that of the control animal. **D**: magnification (25 000×) of an indicated sector of panel **C**, showing small electron-dense aggregates within the lymphocytic cytoplasm (arrows). **E**: Magnification (50 000×) of lymphocytic cytoplasm of the control animal having a very homogenous structure. **F**: Animal exposed to 10 mg/m^3^ SiO_2_.acrylate, magnification 200 000×. Again, the lymphocytic cytoplasm is less homogenous than that of the control animal, and electron-dense particles are found within the cytoplasm. By EDX analysis, the electron-dense particles were identified as being silicon-rich.

In following up the recorded alterations in spleen weights, the *lungs and spleens* from three animals, each, from the control and high concentration groups were investigated by **transmission electron microscopy**. In the lung, electron-dense aggregates consisting of small (approx. 20 nm) particles were recorded in the alveolar space of the exposed animal with no corresponding findings in the control animals (images not shown). In the spleen of the animals treated with 10 mg/m^3^ SiO_2_.acrylate, thrombocyte accumulations were observed (Figure 4B) whereas the spleens of the control animals were unaffected (Figure 4A). Additionally, the cytoplasm of the splenetic lymphocytes seemed to be less homogenous in the exposed animals (Figures 4C and 4D) than in the control animals (Figure 4E), and small electron-dense aggregates were found within the lymphocytic cytoplasm of the treated animals (Figures 4D and 4F). Since silicon particles were detected in the spleen (for details on the outcome of the organ burden analysis, *c.f.* Organ burden analysis), these morphological changes were assessed as being related to the test material, even though the physiological meaning of the findings in the spleen remains unclear.

Overall, the **NOAEC** for local effects in the respiratory tract was assessed as being at least 10 mg/m^3^. Taking into account the findings recorded for the spleen, the **NOEC for systemic effects** was 0.5 mg/m^3^.

##### Other surface-coated amorphous silica (SiO_2_.PEG, SiO_2_.phosphate, and SiO_2_.amino)

*No adverse effects* were observed after inhalation exposure to the three silica particles surface-modified with polyethyleneglycol (SiO_2_.PEG), phosphate (SiO_2_.phosphate), or amino groups (SiO_2_.amino) at concentrations up to 50 mg/m^3^ (Table 3; and Additional file 1: Table S1, Additional file 1: Table S4). Therefore the **NOAEC** for these compounds was assessed as being at least 50 mg/m^3^.

In comparing the effects elicited by the surface-coated silicas with those caused by naked amorphous silica, surface modifications apparently mask the toxicity of the core material.

#### Barium sulfate (BaSO_4_)

Nano-BaSO_4_*did not induce any treatment-related effects* up to an aerosol concentration of 50 mg/m^3^ (Table 3; and Additional file 1: Table S1, Additional file 1: Table S5). Only in the tissue homogenates of the lavaged lungs, a transient increase in IL-1α was observed that was assessed as not being biologically relevant. Therefore, the **NOAEC** for BaSO_4_ was determined as being at least 50 mg/m^3^. These results stand in line with a previous study investigating BaSO_4_ effects upon intratracheal instillation [25], where bolus doses of 4.8 mg per rat lung did not affect any parameters of the BALF. Subchronic inhalation exposure to an even higher concentration (75 mg/m^3^) of micron-scale BaSO_4_, resulting in a lung burden of 5 mg/lung, also did not result in increased PMN counts in the BALF [26]. Whereas the authors attributed this lack of effects to the small surface area of fine BaSO_4_ (3.13 m^2^/g), micron-scale TiO_2_ (with a similar surface area of 6 m^2^/g) elicited pronounced increases of PMN neutrophils in the BALF in the same concentration range [27].

#### Zirconium dioxide-based compounds

##### Nano-zirconium dioxide (nano-ZrO_2_)

Inhalation exposure to aerosol concentrations of up to 10 mg/m^3^ nano-ZrO_2_*did not induce any treatment-related effects* in cytological, protein, enzyme, cytokine or chemokine levels in the BALF or in cytokine levels in the lung tissue (Table 3; and Additional file 1: Table S1, Additional file 1: Table S5), even though the comprehensive panel of 68 cell mediators was assessed both in the BALF and lung tissue (*c.f.* Summary of results). Likewise, the hematological parameters and acute phase protein levels in the blood remained unchanged. There were no histopathological changes of the respiratory tract, and cell proliferation rates and apoptotic reactions in lungs cells were comparable to those from the control groups (data not shown). Thus, the **NOAEC** for nano-ZrO_2_ was assessed as being 10 mg/m^3^.

This finding stands in line with human data for both ZrSiO_4_ and ZrO_2_. In an occupational setting, 32 hand finishers of zirconium metal, who were exposed to 5.75-14.7 mg/m^3^ of dust (25% zirconium) over periods of 1–17 years, did not develop any exposure-related symptoms [28]. Similarly, a study following up zirconium compound-exposed workers revealed that even long-lasting exposure of up to 20 years to peak concentrations up to 30 mg/m^3^ zirconium neither elicited abnormal chest radiographs, nor did it impair lung function parameters [29]. Hence, the lack of effects of nano-ZrO_2_ observed in the present study may be due to the very low intrinsic toxicity of this material.

##### Surface-coated zirconium dioxide (ZrO_2_.TODA, and ZrO_2_.acrylate)

*No adverse effects* were observed after inhalation exposure to the surface-coated zirconium dioxides ZrO_2_.TODA or ZrO_2_.acrylate at concentrations up to 50 mg/m^3^ (Table 3; and Additional file 1: Table S1, Additional file 1: Table S5). Under the conditions of the present study, the **NOAEC** for these compounds was therefore at least 50 mg/m^3^.

#### Cerium dioxide-based materials

##### Nano-cerium dioxide and Al-doped nano-cerium dioxide

Overall, nanoform cerium dioxide (nano-CeO_2_) and Al-doped nano-CeO_2_ elicited similar effects with respect to BALF cytology and mediators.

At aerosol concentrations of 0.5 mg/m^3^*nano-CeO*_*2*_, PMN neutrophil counts, total protein concentration and macrophage colony stimulating factor (M-CSF) levels were increased in the **BALF**. Inhalation exposure to 2.5 and 10 mg/m^3^ nano-CeO_2_ increased PMN neutrophil and lymphocyte counts in the BALF immediately after the final exposure, whereas macrophage counts were reduced in comparison to the corresponding control values (Table 3; Figures 3E and 3F; and Additional file 1: Table S1, Additional file 1: Table S6). Several enzyme activities (LDH, GGT, ALP) were increased in the BALF in the 2.5 and 10 mg/m^3^ exposure groups, whereas NAG activities and total protein levels were only increased at 10 mg/m^3^. Among the 68 antigens assessed in the BALF, the level of monocyte chemoattractant protein-1 (MCP-1) was prominently increased (approx. 20- to 25-fold over the corresponding control values), while M-CSF and CINC-1 levels were only moderately elevated (below 10-fold). The macrophage markers macrophage-derived chemoattractant (MDC) and myeloperoxidase (MPO) were increased 360-fold and 115-fold, respectively. After the three-week post-exposure period, a partial regression of these effects was observed (Table 3; Figures 3E and 3F; and Additional file 1: Table S1, Additional file 1: Table S6).

Also in the **lung tissue homogenates**, of the 68 antigens assessed, 9 cytokines and chemokines (i.e. CINC-1/IL-8; keratinocyte cytokine/growth-regulated oncogen-α (KC/GROα); MCP-1; MCP-3; M-CSF; MDC; macrophage inflammatory protein (MIP-1α); MIP-2; neutrophil gelatinase associated lipocalin (NGAL)) were significantly increased in the 2.5 and 10 mg/m^3^ nano-CeO_2_ exposure groups, and increases only partially regressed within the post-exposure period (Additional file 1: Table S1, Additional file 1: Table S7).

*Al-doped nano-CeO*_*2*_ also induced significant increases in total cell counts, PMN neutrophil counts, and total protein concentration, and elevations in all enzymes tested. In the BALF, Al-doped nano-CeO_2_ further elicited higher concentrations of OPN than nano-CeO_2_, but lower levels of M-CSF, and, in the lung tissue, higher levels of IL-1α (nano-CeO_2_ data for OPN and IL-1α, being insignificant, not shown). The extent of partial regression of the elevated lung parameters was comparable in the recovery groups treated with either nano-CeO_2_ or Al-doped CeO_2_ (Table 3; and SI, Additional file 1: Tables S8 and S9).

Hematology parameters and acute phase protein levels in the **blood** were not affected in rats treated with either CeO_2_ compound. **Histological examination** of the *nano-CeO*_*2*_ test groups revealed particles in alveolar macrophages in the exposure and recovery groups and, additionally, in the recovery groups, mild diffuse or multifocal alveolar histiocytosis. In the exposure groups treated with *Al-doped nano-CeO*_*2*_, single or aggregated particle-loaded alveolar macrophages were observed that were still present after the post-exposure period, though less frequently (SI, Additional file 2: Tables S14 and S15). **Cell proliferation rates and apoptosis** in rats exposed to nano-CeO_2_ were comparable to the rates recorded in the corresponding control groups (data not shown).

Overall, inhaled nano-CeO_2_ and Al-doped nano-CeO_2_ caused a transient, concentration-dependent inflammation of the lung at all concentrations. Based upon the changes recorded in the BALF, the concentration-response curve of Al-doped CeO_2_ was steeper than the one calculated for nano-CeO_2_. *In vitro* studies with alveolar macrophages performed within the German NanoCare project determined a higher biological activity for Al-doped CeO_2_ than for nano-CeO_2_[25], an observation that is in agreement with the *in vivo* STIS results of the present study. Taking into account the increased PMN neutrophil counts in the BALF, a **NOAEC** could not be established for nano-CeO_2_ or Al-doped nano-CeO_2_ (NOAEC < 0.5 mg/m^3^).

These results stand in line with the outcome of a previous study investigating CeO_2_ effects upon intratracheal instillation of [16], showing that already bolus doses as low as 0.15 mg/kg body weight – corresponding to the low aerosol concentration of 0.5 mg/m^3^ applied in the present STIS - affected BALF parameters.

### Organ burden analysis

The **lungs** (Table 4) and **mediastinal lymph nodes** (data not shown) were examined for the contents of the respective main element of the test material. For most test substances, the recorded pulmonary deposition was consistent with the expected deposition calculated making use of the Multiple Path Particle Dosimetry Model (MPPD software, version 2.11) [30,31]. The decrease in lung burden during the post-exposure period was around 20% for most test substances, reflecting a clearance rate with a half-life of about two months. For nano-ZrO_2_, BaSO_4_, the SiO_2_ test substances (apart from SiO_2_.acrylate), micron-scale ZnO and coated nano-ZnO, the decrease rates were markedly higher: For nano-ZrO_2_, decreases of up to 75% were observed. In animals exposed to BaSO_4_, the lung burden decreased by 77% during the three-week post-exposure period without notable increase of the substance in the lung-draining lymph nodes. Amorphous silica seemed to be cleared quickly as well: For naked amorphous silica, SiO_2_.PEG, and SiO_2_.amino, about 40 to 60% of the total deposition were cleared within 3 weeks. Unlike the PEG and amino coatings, however, the polyacrylate coating seemed to hamper the clearance rate of SiO_2_. The most pronounced decrease was recorded for those animals exposed to nano- or micron-scale ZnO: After the post-exposure period, the measured Zn content returned to the control level in all test groups. This observation can be explained by the dissolving properties of zinc oxide.

Overall, only very small amounts of the test substances were found in the **lung-draining lymph nodes** (data not shown). In animals exposed to either of the ZrO_2_ materials, zirconium was not detectable at any time point in any of the examined animals. In animals exposed to nano-CeO_2_ or Al-doped nano-CeO_2_, cerium levels were below the detection limit in the exposure groups. In the recovery group, they were 1.4, 2.5, and 4.1 μg nano-CeO_2_, respectively, for the 3 examined rats exposed to 10 mg/m^3^ (effective concentration 11.6 mg/m^3^) of this test material, and 2.2 μg for one animal treated with 10 mg/m^3^ (effective concentration 9.2 mg/m^3^) Al-doped CeO_2_. Similarly, barium was not detectable in the lymph nodes of the exposure groups and was only 1.4 μg in the high concentration recovery group exposed to 50 mg/m^3^ BaSO_4_. Comparable amounts of zinc were found in the lung-draining lymph nodes regardless of the applied test concentration or the time point of assessment. Upon inhalation exposure to nano-TiO_2_, test substance translocation to the lung-draining lymph nodes was much lower than it had previously been observed after exposure to high concentrations of micron-scale TiO_2_ or quartz [27].

In those animals exposed to coated nano-TiO_2_, BaSO_4_, Al-doped CeO_2_, nano- or micron-scale ZnO, the **liver, kidneys, spleen, and brain (including the olfactory bulb)** were also examined for the content of the respective main element, with a detection limit of 500 ng/tissue sample (data not shown). In all organs evaluated, the titanium content was below this detection limit. Hence, there was no indication for test substance translocation upon inhalation exposure to coated nano-TiO_2_. The contents of barium or cerium detected in the liver, as the only extra-pulmonary organ, might be associated with BaSO_4_ or CeO_2_ dissolution in the body [26,32]. Also zinc oxide is known to be soluble in the body [33]. Therefore some degree of transport of zinc ions to peripheral organs was to be expected. Notwithstanding, the background level of Zn was high in all animals of the control group (20 μg/lung; 300 μg/liver), and the Zn content determined in the organs of the animals treated with coated nano-ZnO and micron-scale ZnO was indistinguishable from the respective background levels determined in the control animals (data not shown).

As regards translocation of inhaled SiO_2_.acrylate to extra-pulmonary organs, small particle aggregates (60–80 nm) were present in the cytoplasm of lymphocytes of the white pulp of rats exposed to 10 mg/m^3^ SiO_2_.acrylate (as evidenced by TEM; Figures 4C, 4D, 4F; *c.f.* Amorphous silica-based materials). Using energy dispersed x-ray spectroscopy (EDX), these particles were identified as being silicon-rich, whereas the surrounding cytoplasm contained only low background levels of silicon (Figure 4F).

### Summary of results

In summary, the present study investigating the effects of 13 nanomaterials or micron-scale ZnO upon inhalation exposure to rats at aerosol concentrations of typically 0.5 to 50 mg/m^3^ for five consecutive days (6 h/day) revealed the following findings (Table 3):

● Eight nanomaterials (BaSO_4_, SiO_2_.acrylate, SiO_2_.PEG, SiO_2_.phosphate, SiO_2_.amino, nano-ZrO_2_, ZrO_2_.TODA and ZrO_2_.acrylate) did not elicit effects on the rat lung, and their **(local pulmonary) NOAECs were at least 50 mg/m**^**3**^ (or at least 10 mg/m^3^ if this was the highest concentration tested).

● SiO_2_.naked, multifocal macrophage aggregates were observed in the respiratory tract immediately after the exposure period that exacerbated towards a slight multifocal inflammation during the 3-week post-exposure period. Its **NOAEC was 2.5 mg/m**^**3**^.

● Four nanomaterials (coated nano-TiO_2_, coated nano-ZnO, nano-CeO_2_, Al-doped nano-CeO_2_) evoked transient and concentration-dependent pulmonary inflammatory reactions that were only partially reversible during the two- or three-week post-exposure period. Their **NOAEC was 0.5 mg/m**^**3 **^**or below**. The same applies to micron-scale ZnO, for which, however, no NOAEC was laid down since it was only tested at one (high) test substance concentration.

● Observed **extra-pulmonary effects**, immediately after the final exposure, were the **splenetic alterations** recorded for SiO_2_.acrylate (for which therefore a systemic NOEC was set at 0.5 mg/m^3^) and the moderate to severe **necrosis of the olfactory epithelium** recorded for micron-scale ZnO and coated nano-ZnO. These splenetic effects were fully reversible within the post-exposure period, and the nasal cavity alterations partially reversible.

## Discussion

In 2009, the short-term inhalation study protocol (STIS) was established to fulfil the urgent need for a rapid and reliable method to evaluate the pulmonary toxicity of nanomaterials [1]. The protocol was based on the hypothesis that a nanomaterial’s toxicity (1) is initiated by its inflammatory potential and (2) is influenced by its deposition and translocation in the body and its clearance therefrom. Both aspects can be determined after a relatively short exposure period when including specific investigations of the animals. Meanwhile, more than 20 industrially relevant nanomaterials have been tested in the STIS, covering the 13 nanomaterials presented in this study, four carbon-based nanomaterials [34,35], two cadmium-based quantum dots [36,37], one polyacrylate [38], and one pigment [Ma-Hock and co-workers, unpublished observations]. In the following section, the experiences gained with all STIS conducted so far are assessed with the aim of re-evaluating the study design of the STIS and of determining its relevance in predicting nanomaterial effects that develop upon subchronic or chronic exposure.

### Study protocol

The study design for the current STIS was first endorsed based on an evaluation of an extensive data set for nano-TiO_2_[1]. Since then, the key elements of the study design have remained unchanged, i.e.:

● Male Wistar rats;

● Head-nose inhalation exposure;

● Exposure for six hours a day on five consecutive days;

● Histological examination and bronchoalveolar lavage;

● Post-exposure period of three weeks.

With regard to histological examination, experience has shown that it is essential to evaluate the entire respiratory tract. First of all, lavage parameters only reveal pulmonary effects. Hence, effects occurring in the upper respiratory tract might be missed without histological examination. Coated nano-ZnO and micron-scale ZnO are examples for substances affecting the upper respiratory tract. Additionally, classical BALF parameters are general indicators of acute inflammatory reactions, which are not necessarily associated to a specific type of morphological change. For all of the above-mentioned nanomaterials tested in the STIS so far, the pattern of BALF changes has been similar: The number of PMN neutrophils was the most sensitive parameter and was accompanied by increases of other BALF cells, increased BALF total protein concentrations and increased enzyme activities. Notwithstanding these very similar BALF findings, the spectrum of different nanomaterials investigated elicited clear differences in morphological changes of the lung tissue, to name multiwall carbon nanotubes (MWCNTs) as a prominent example [34,35]. Consequently, histological examination of the entire respiratory tract should be an integral part of the STIS protocol.

In *in vitro* studies, pro-inflammatory cytokines are being widely applied as indicators of acute or on-going inflammation or inflammation-like responses. Additionally, pro-fibrotic cytokines, such as transforming growth factor (TGF)-β1, M-CSF, or OPN, may be useful in identifying particle-induced fibrotic or neoplastic changes already at very early pathogenic stages. Aiming at recognizing appropriate markers for *in vivo* studies, such as the STIS, almost 70 different cytokines, chemokines and inflammation-relevant enzymes from the BALF and lung tissue homogenates have been screened in the course of the development of the STIS. Of these mediators, only a limited panel appear to be relevant for the assessment of particle effects in the lungs. Specifically, 4 mediators, i.e. MCP-1, CINC/IL8, M-CSF and OPN, were selected as being most meaningful for the detection of substance-induced pulmonary reactions in the BALF.

**MCP-1 and CINC-1/IL-8**, both of which are released by epithelial cells and, to a certain extent, also by macrophages, reached considerable concentrations in the BALF upon particle treatment. MCP-1 is a C-C cytokine that strongly attracts blood monocytes and lymphocytes to the alveolar compartment [39]. CINC-1, the rat homologue to IL-8, is a pro-inflammatory C-X-C chemokine that exhibits neutrophil chemotactic activity [40]. Dose-dependent increases of MCP-1 and CINC-1/IL-8 in the BALF were recorded upon inhalation exposure to nano-CeO_2_, Al-doped nano-CeO_2_, coated nano-ZnO, or micron-scale ZnO. Apart from the dose groups treated with coated nano-ZnO, the increases in MCP-1 exceeded those of CINC-1/IL-8.

Reporting increased IL-8 expression in human A549 alveolar epithelial cells upon exposure to micron-scale BaSO_4_ (and similar observations published by Tran et al. [13]), Donaldson et al. [40] suggested IL-8 induction as being a relevant parameter for the validation of *in vitro* assays. However, in contrast to the observations made by Donaldson and co-workers, in the present study, up to 50 mg/m^3^ nano-BaSO_4_ did not lead to any biologically relevant cytokine induction. This discrepancy might be explained by differences of the BaSO_4_ materials or by deviating experimental regimes. Nevertheless, it should be followed up in further studies aiming at correlating *in vivo* responses to *in vitro* IL-8 production in epithelial cells. Regardless of any possible *in vitro-in vivo* discrepancies, both MCP-1 and CINC/IL-8 displayed a large dynamic range in the present study, and hence were assessed as suitable markers for early pulmonary inflammation and macrophage recruitment to the lungs.

**M-CSF** is a cytokine produced by macrophages that is involved in the differentiation of monocytes into histiocytes or, in conjunction with other factors, in the differentiation of osteoclasts [41]. At least in mice, its over-expression has been associated with glycolipid-induced granulomas [42], and M-CSF might possibly induce an increase in the number of alveolar macrophages. In the present study, M-CSF levels were not elevated upon exposure to nano-BaSO_4_, or polyacrylate-coated amorphous silica, while pronounced M-CSF increases were elicited by coated nano-ZnO and micron-scale ZnO, and nano-CeO_2_. However, the M-CSF concentrations in the BALF did not correlate with macrophage counts in the BALF, presumably due to the lavage technique used, allowing only small fractions of alveolar macrophages to be washed out.

**OPN** is an arginine-glycine-aspartic acid (RGD)-containing protein occurring not only in the extracellular matrix of mineralized tissues, but also as a cytokine in body fluids [43]. OPN transcription is, among others, stimulated by IL-1 and/or TNF-α. As a cytokine, OPN has both pro- and anti-inflammatory properties. With respect to the latter, it inhibits production of nitric oxide (NO) and down-regulates inducible NO synthase [44]. More specifically, OPN has been described to be involved in pulmonary granuloma formation in rodents [45,46]. In rats exposed subchronically to 50 mg/m^3^ micron-scale TiO_2_, a concentration eliciting lung inflammation and fibrosis [7], immediate and sustained formation of OPN was observed. These and other results strongly suggest that OPN is a useful biomarker for fibro-proliferative lung disease in rodents and humans [47].

In the present study, OPN was increased up to 10-fold directly after inhalation of nano-CeO_2_, coated nano-ZnO, and micron-scale ZnO. In all of these cases, OPN increases were transient, and they were accompanied by elevations of other pro-inflammatory cytokines, such as MCP-1 or IL-1α. Even though the corresponding histological examinations revealed no signs of beginning granulomatous changes or fibrosis, Cho and co-workers [9] recorded the development of granulomas and deposition of fibrotic tissue, respectively, upon instillation of CeO_2_ or ZnO nanoparticles. By contrast, the slight increase in OPN observed after the three-week post-exposure period in the recovery group treated with SiO_2_.naked was not accompanied by changes in any other cytokines and, therefore, was assessed as being arbitrary.

Aiming at supplementing evaluation of the BALF, the added value of determining **TNF-α and IL-1α** as lung tissue homogenate parameters was assessed. TNF-α and IL-1α are pro-inflammatory cytokines released by activated macrophages and other immune cells. By autocrine and/or paracrine pathways, they can stimulate release of further chemokines from other macrophages or epithelial cells [48-50]. TNF-α is a pleiotropic cytokine and a strong chemoattractant for PMN neutrophils [50,51]. Therefore it contributes to the early PMN neutrophilic infiltration and eosinophil recruitment into the airways, and it further increases alveolar capillary permeability [51,52]. In previously performed STIS, both IL-1α and TNF-α were found in higher concentrations in lung tissue homogenates than in the BALF. This may be explained by IL-1α also being synthesized by epithelial and endothelial cells and/or by the observation that approximately 80% of the pulmonary macrophages remain in the lung after the first lavage [53]. In all STIS performed in the course of the present study, only small and inconsistent increases in TNF-α or IL-1α levels were observed in the BALF or lung tissue homogenates, and these minor changes were found to add only limited information to the understanding of substance-induced early inflammatory processes occurring in the lung.

Similarly, also the assessment of cell proliferation rates or apoptosis in the large and medium bronchi, terminal bronchioli, or alveoli did not provide substantial added value information for the toxicological assessment of the test materials investigated in the present study.

### Correlation of the STIS with chronic or subchronic inhalation studies

In an extensive case report by Klein and co-authors [3], the results of all STIS that were available at the time of writing in 2012 were compared to the results of subchronic and chronic inhalation studies of the same or similar materials. The review also included the overall results of a few of the STIS presented and discussed in detail in the study at hand. The list of materials assessed by Klein and co-authors comprises commercially relevant nanomaterials and selected non-nanoform reference materials, i.e. quartz, pigmentary and nanosized TiO_2_, synthetic amorphous silica, zinc oxide, zirconium dioxide, and multiwall carbon nanotubes. The studies included in the case report were not identical in regard to experimental conditions, such as animal strains, physico-chemical characteristics of the test materials, or preparation of the test material. Furthermore, only a limited number of subchronic and chronic studies were available against which to compare the results of the STIS. Nevertheless, the ranking of the spectrum of nanomaterials in regard to their potential to induce adverse effects was concordant for the NOAECs determined in the STIS and in the corresponding subchronic and chronic studies, and it was also consistent with the ranking set up in the present study. Furthermore, the STIS were able to reveal the progression or regression of adverse effects, to indicate translocation of materials to extra-pulmonary organs and to provide early indications of effects that only develop over time and hence only become fully pronounced in longer-term studies [3].

Overall, the STIS is able to detect *early* effects that mostly reflect inflammation, and/or beginning histological changes, while a meaningful comparison of subchronic toxicity endpoints, such as fibrosis and cancer, to STIS parameters is hardly possible. Nevertheless, sustained inflammation is being suspected to be a necessary, though not sufficient, prerequisite for enhanced reactive oxygen species (ROS) formation, fibrosis and even cancer, at least in the rat lung [54]. Therefore, an estimation of inflammatory effects at an early stage (directly after the final exposure or two days thereafter) combined with data on reversibility of the effects (three weeks post-exposure) may be used as an indicator for the severity of toxic effects.

Taking into account all of these considerations, the STIS is assessed as a suitable test method to be applied as a versatile first step in a tiered testing approach. As an early tier test, the STIS enables prioritizing nanomaterials for further testing and allows selecting appropriate testing strategies for the given test substance, which may, or may not, include subchronic or chronic tests. In the report of the NanoSafety Cluster Working Group 10, application of a targeted strategy for the testing and assessment of nanomaterials was encouraged, and the STIS was suggested as a basic test for Tier 2, i.e. the first tier requiring testing, upon collection of all available information in Tier 1 [55].

### Application of the STIS data for the grouping of nanomaterials

The STIS provides information on the lung burden, test substance translocation to extra-pulmonary organs, as well as the type and potency of effects in the lung and in other tissues together with the persistence of these effects. Making use of these elements of the STIS protocol, the 14 materials (13 nanomaterials) assessed in the present study can be grouped in accordance to (1) the potency of pulmonary inflammation; (2) the affection of extra-pulmonary organs; (3) the reversibility or persistence of effects.

#### Nanomaterial grouping based on the potency of lung inflammation

**Figure 5:**
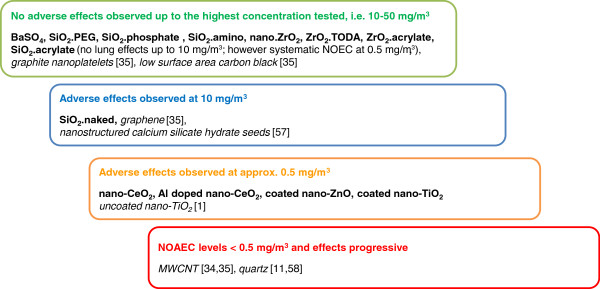
**Ranking of nanomaterials according to their toxic potency.** Names of substances presented in this paper are printed in bold. Names of substances assessed in other studies are printed in italics and the corresponding reference is provided [1,11,35,57,58].

Based on the pulmonary effects observed at different aerosol concentrations in the STIS, the test materials evaluated in the present study (and in previously performed STIS) can be assigned to four different potency groups (Figure 5):

● Substances causing no lung effects in the STIS up to aerosol concentrations of 50 mg/m^3^, i.e. SiO_2_.PEG, SiO_2_.phosphate, SiO_2_.amino, BaSO_4_, nano-ZrO_2_, ZrO_2_.TODA, ZrO_2_.acrylate. In further STIS, graphite nanoplatelets and carbon black did not induce any adverse effects up to the highest test concentration 10 mg/m^3^[35]. SiO_2_.acrylate did not cause any pulmonary effects up to the highest concentration of 10 mg/m^3^, but it did elicit splenetic effects at 2 mg/m^3^ and above, so that a systemic NOEC of 0.5 mg/m^3^ was determined for this substance.

● Substances causing lung effects in the STIS at aerosol concentrations at 2.5 mg/m^3^, i.e. precipitated naked amorphous silica;

● Substances causing lung effects in the STIS at aerosol concentrations of approximately 0.5 mg/m^3^, i.e. uncoated nano-TiO_2_[1], coated nano-TiO_2_, ZnO, coated nano-ZnO, nano-CeO_2,_ Al-doped nano-CeO_2_;

● Materials causing lung effects in STIS at approximately 0.1 mg/m^3^: None of the 14 test materials applied in the present study was tested at such low aerosol concentrations, since they were not expected to possess a very high toxic potency. For MWCNTs, however, a NOAEC at or below 0.1 mg/m^3^ was calculated in a STIS, and this very low value was subsequently confirmed in two unrelated subchronic inhalation studies [35,56].

Of note, this STIS-based potency ranking is largely consistent with a banding scheme suggested by the US National Institute for Occupational Safety and Health (NIOSH) [59].

Based on the toxic potency information obtained in the STIS, CeO_2_ and BaSO_4_ nanomaterials were selected for 2-year combined chronic toxicity/carcinogenicity inhalation studies that are still ongoing (OECD TG 453).

#### Nanomaterial grouping based on affected extra-pulmonary organs

Of the 13 nanomaterials tested in the STIS in the present study, only two caused effects outside the lung. These were the same materials that were also detected in extra-pulmonary tissues, i.e. polyacrylate-coated SiO_2_ and coated nano-ZnO. Inhalation of SiO_2_.acrylate resulted in enlarged spleens, and ZnO caused damage of the upper airways. These findings confirm the value of collecting tissue burden data, and affection of extra-pulmonary organs may also (and correspondingly) be used as a parameter for grouping and testing strategies. Based upon the results of the STIS, further testing of these materials should include effects on target organs.

#### Nanomaterial grouping based on the reversibility or persistence of effects

The pulmonary effects observed upon rat 5-day inhalation exposure to coated nano-TiO_2_ or the ZnO and CeO_2_ compounds were not fully reversible within the three-week post-exposure period, whereas the systemic effects caused by polyacrylate-coated SiO_2_ were no longer observed after this period. One reason for effects being only partially regressive might be that the three-week post-exposure period was not sufficiently long to allow full recovery to take place, such as is most likely the case for the soluble ZnO materials. Particle solubility presumably is an important factor determining reversibility of effects. Consequently, e.g., amorphous silica nanomaterials having some (low) degree of solubility would be less prone to elicit long-term effects than TiO_2_ nanomaterials for which particle dissolution has not been recorded.

Alternatively, partial regression of effects might be an indication that the development of chronic effects cannot be excluded, especially if the lung clearance mechanism is overloaded. Such specifications obviously provide valuable information for the design of further investigations of these substances. Moreover, information on the reversibility or persistence of effects, combined with substance pulmonary clearance rates, can be used as a grouping criterion for hazard assessments addressing short-term exposure scenarios. Likewise, progressive pulmonary alterations, such as fibrosis upon inhalation exposure to quartz or the formation of granuloma upon exposure to MWCNTs [34,35], fall into this category.

## Conclusion

Short-term inhalation studies with aerosols generated from 14 particulate materials (1 micron-scale and 13 nanomaterials) provided information on the biokinetics and pulmonary and extra-pulmonary effects of the test materials. It further allowed ranking the materials in regard to their potency based upon NOAECs that were derived from well-quantifiable parameters, such as PMN neutrophil counts or total protein concentrations in the BALF, or from the incidence and severity of histological findings (Figure 5).

A number of the test materials did not elicit adverse pulmonary effects under the experimental conditions of the present study, i.e. BaSO_4_, all ZrO_2_ materials and the surface-functionalized SiO_2_ materials. With the exception of SiO_2_.acrylate, also no systemic effects were observed for these substances. Hence, the outcome of the present study points to the low pulmonary toxicity of these nanomaterials upon inhalation.

Apart from serving to reveal a substance’s toxic potency, information on the type, location and reversibility of the observed effects can be used to guide further testing, e.g. in regard to those materials for which adverse pulmonary effects (nano-TiO_2_, both CeO_2_ nanomaterials and SiO_2_.naked) or adverse extra-pulmonary effects (both ZnO materials and SiO_2_.acrylate) were observed.

Additionally, the results from the STIS can be used for the grouping of nanomaterials for safety assessment. It is increasingly being recognized that it is not possible to relate biological effects elicited by nanomaterials to one single material property and that other forms of grouping might be more relevant in predicting nanomaterial effects [55,59]. Groups of nanomaterials with similar toxicity can be defined based upon similarities in biological activities, lung tissue burden or translocation to extra-pulmonary tissues, rather than merely referring to the materials’ properties. In this context, the STIS data published so far provide a large database on effects upon inhalation exposure to nanomaterials that can serve to foster the understanding of material properties, which are attributable to toxic effects of nanoparticles.

Based upon the overall outcome of the present study, the STIS is suggested as an early tier test in an integrated approach to the testing and assessment of nanomaterials. As the comparison of the outcome of the STIS with results from subchronic and chronic inhalation studies has revealed, the STIS may well be suited in reducing the need for such longer-term studies. Furthermore, the STIS may be supplemented or even replaced by appropriate *in vitro* methods once they become available. In this respect, STIS data will contribute to the development and validation of such *in vitro* methods, thereby directly serving the 3Rs principle to refine, reduce, and, ultimately, replace animal testing as first described by Russell and Burch in 1959 [60] and laid down as legal provision in the European Directive 2010/63/EU on the use of animals for scientific purposes [61].

## Material and methods

### Test materials

As presented in Table 1, the characterization of the material properties included particle size distribution – in the dry state and in relevant media; state of agglomeration or aggregation, water solubility and dispersability, crystalline phase, crystallite size, specific surface area, zeta potential (surface charge), surface chemistry (where appropriate), photocatalytic activity and porosity. The applied characterization methods have previously been described in detail together with a discussion of the results of the physico-chemical characterization for the powders [22] and the nanoGEM project suspensions [23] (Table 5).

### Aerosol generation and monitoring

Liquid nanomaterial dispersions were delivered at a constant rate into an atomizer and aerosolized by compressed air. For solid materials, dust aerosols were produced by dry dispersion of powder pellets with a brush dust generator (developed by the Technical University of Karlsruhe, Germany, in cooperation with BASF, Germany). The different target concentrations were achieved by adjustment of the feeding speed of the substance pellet and the rotation speed of the brush. Aerosols were generated with compressed air and diluted with conditioned air. The aerosols were passed via a cyclone (to separate particles > 3 μm) into the head-nose inhalation system. To reduce electrostatic charging, brushes made of stainless steel were used, and the generator itself and all conducting tubes were grounded [57,62].

The atmospheric concentration of the aerosols and the aerodynamic particle diameter (cascade impactor analysis) were determined by gravimetric measurement using a sampling probe with a diameter of 7 mm. In general, the sampling velocity was adjusted to 1.25 m/s, and the sampling flow rate was 3 L/min. Under these conditions, the sampling error due to an-isokinetic sampling is considered to be negligible [62].

To determine the atmospheric aerosol concentration, a sampling device (Millipore) equipped with glass fibre filters (Macherey-Nagel, Düren, Germany, type MN 85/90 BF, d = 4.7 cm) was used. Sampling was performed adjacent to the location where the animals’ noses were positioned in the exposure system. Depending on the respective atmospheric concentrations, the sample volumes ranged from 10–360 L to achieve a sample mass of 1–5 mg per sample. The aerosol concentration was calculated from the difference between the weight of the pre-weighed filter (Sartorius M3P-000 V001 balance; Sartorius, Germany) and the weight of the filter after sampling with reference to the sample volume of the aerosol. Two samples were drawn from each exposure chamber on each exposure day (total 10 samples per concentration over the 5-day exposure period). Means and standard deviations were calculated from these 10 samples per concentration.

Aerosol particle characterization was performed with an eight-stage **Cascade Impactor** (Marple Personal Cascade Impactor; Sierra-Andersen, USA). The effective aerodynamic cut-off diameters were 21, 15, 10, 6.5, 3.5, 1, 0.7, or 0.4 μm. A backup filter was used to collect particles smaller than 0.4 μm. A vacuum pump (Millipore; Merck, Darmstadt, Germany) with a flow-limiting orifice calibrated to 3 L/min (Millipore) was used for sampling. The stages were weighed before and after sampling using a Sartorius balance (see above) or a Mettler AT 250 balance (Mettler-Toledo, Germany). Sample volumes were between 15 L and 720 L depending on their atmospheric concentration.

Additionally, particle size distribution was measured with a **Scanning Mobility Particle Sizer** (SMPS, model 3022A/3071A; TSI, USA). The SMPS system comprised a Model 3071A Electrostatic Classifier separating the particles into known size fractions and a model 3022A Condensation Particle Counter measuring particle count concentrations. Particles in the size range from 0.015-0.805 μm were measured with 32 size channels per decade. The SMPS sampled at a frequency of 0.3 L/min, and the sheath flow was 3 L/min. At this setting, larger particles were removed by an impactor (0.8 μm cut-off) at the inlet of the SMPS.

Finally, all aerosols were measured with an **aerosol spectrometer** (WELAS 2000; Palas, Karlsruhe, Germany) in which single particles are illuminated with a white-light source. The measuring range of the sensor was 0.3-10 μm and the sampling flow rate 5 L/min.

### Animals

Permission for performance of the animal studies was obtained from the local regulatory agencies, and all protocols stood in compliance with the respective federal guidelines. Male Wistar (strain Crl:WI (Han)) rats (7 weeks of age; specific pathogen free) were obtained from Charles River Laboratories (Sulzfeld, Germany). The laboratories of BASF’s Experimental Toxicology and Ecology Unit, where all studies were performed, possess an AAALAC accreditation (International Association for Assessment and Accreditation of Laboratory Animal Care). All procedures for animal care and exposure were conducted in accordance with the provisions of the German Animal Welfare Act (1998). For a few substances, the animals were housed individually in mesh-floored cages, whereas for the majority of the studies (that were performed at a later time point), social housing was applied (polysulfonate cages (H-Temp [PSU]), floor area about 2065 cm^2^ (610×435×215 mm); supplied by TECHNIPLAST, Germany). All animals rooms were maintained at 20-24°C, with a relative humidity of 30-70% and a light/dark cycle of twelve h each. All animals were acclimatized to these conditions for about two weeks before the onset of the study. In home cages, the animals were allowed free access to laboratory diet (Provimi Kliba SA, Basel, Switzerland) and water, but not during the exposure periods.

### Study design

The male Wistar rats (*c.f.* Table 5 for the concrete numbers of animals used for the testing of the different materials) were exposed to test substance aerosols, or conditioned air in the control groups, for six h per day on five consecutive days by head-nose exposure. **‘Exposure groups’** were euthanized shortly after the final exposure, and **‘recovery groups’** after the three-week post-exposure period (two weeks for SiO_2_.acrylate).

Bronchoalveolar lavage of 5 rats/test group was performed, and the **BALF** was analysed, determining BALF cytology, total protein, enzyme activities and (apart from the test groups treated with coated nano-TiO_2_) BALF cytokines and chemokines (described in detail in Evaluation of bronchoalveolar lavage fluid (BALF) and lung tissue homogenates). For a number of test substances, cytokines and chemokines were additionally assessed in **tissue homogenates of the lavaged lungs** (Evaluation of bronchoalveolar lavage fluid (BALF) and lung tissue homogenates). The **blood** obtained from the 5 animals/test group used for BALF collection (again apart from coated nano-TiO_2_) was submitted to haematology and clinical chemistry in accordance with the parameters listed in OECD TG 412, and, additionally, c-reactive protein and haptoglobin were assessed (Blood analysis). Three or six animals from all test or control groups from the exposure or recovery groups were submitted to **necropsy** and **histopathological evaluation** of the respiratory tract (Necropsy and histopathological evaluation). For those test groups treated with micron-scaled or coated nano-ZnO, nano-CeO_2_ or nano-ZrO_2_, **cell proliferation and apoptosis rates** were determined in the large and medium bronchi, terminal bronchioli, and alveoli (Assessment of cell proliferation and apoptosis). In those animals submitted to necropsy, **organ burden** was determined, i.e. the content of the test materials in the lung, in the mediastinal lymph nodes, and in the extra-pulmonary organs (Organ burden analysis and TEM and EDX organ analysis). In addition, the spleens of 3 rats, each, of the highest dose group treated with SiO_2_.acrylate as well as of the corresponding control group were submitted **to transmission electron microscopic** investigation and, for one spleen, each, to **EDX analysis** (Organ burden analysis and TEM and EDX organ analysis).

The 13 nanomaterials and micron-scale ZnO were tested over a period of four years with slightly differing study designs for the different test materials (*c.f.* Table 5 for details on the study designs for each material and a specification of the respective examinations performed at different time points).

### Evaluation of bronchoalveolar lavage fluid (BALF) and lung tissue homogenates

The animals used for **BALF collection** were anesthetized and exsanguinated, and the exposed lungs were washed by two instillations of physiologic saline solution (flow rate 12 ml/min; instillation volumes were adjusted to the mean group body weight (0.9% (w/v), 6–8 ml/wash). About 92% of the instilled solution was recovered as lavage fluid per animal. The two washes were combined and mixed prior to analysis. Aliquots of combined BALF were used to determine total protein concentration, total cell count, differential cell count, and activities of BALF enzymes as well as mediator levels.

**Total BALF cell counts** were determined with an Advia 120 (Siemens Diagnostics, Fernwald, Germany) hematology analyzer. Counts of macrophages, PMN neutrophils, lymphocytes, eosinophils, monocytes and atypical cells were performed on Wright-stained cytocentrifuge slide preparations as described by [32]. The **differential BALF cell count** was evaluated manually by counting at least 400 cells per sample.

The following parameters were measured with a Hitachi 917 (Roche Diagnostics, Mannheim, Germany) reaction rate analyzer:

● **Total protein** (turbidimetric method with Benzethonium chloride);

● **Lactate dehydrogenase** (EC 1.1.1.27; kinetic UV test, 340 nm, 37°C acc. to the International Federation of Clinical Chemistry (IFCC) reference system of enzymes);

● **Alkaline phosphatase** (EC 3.1.3.1; kinetic color test, 450 nm, 37°C acc. to IFCC);

● **N-Acetyl-β-D-glucosaminidase** (EC 3.2.1.30; color test, 580 nm, 37°C; [63]);

● **γ-Glutamyltransferase** (EC 2.3.2.2, Szasz method (kinetic color test), 415 nm, 37°C acc. to IFCC).

Aiming at determining cell mediators in the BALF that were suitable in predicting substance-induced pulmonary effects, an extensive panel of altogether **68 cytokines, chemokines and inflammation-relevant hormones and enzymes** were assessed in the **BALF** from the test groups treated with micron-scale ZnO, coated nano-ZnO, nano-CeO_2_ or nano-ZrO_2_.

This panel of mediators comprised *67 antigens* that were assessed at Rules-Based Medicine, Inc. (Austin, TX, USA) making use of the xMAP technology (Luminex Corp., Austin, TX, USA) as described previously [32,64-67]. These 67 antigens encompass the 58 antigens of the Rodent Multi-Analyte Profile (RodentMAP® v. 3.0) and 9 additional antigens of the Rat KidneyMAP® v. 1.0 (*c.f.*https://rbm.myriad.com/products-services/rodentmap-services/ for full lists of the respective panels of mediators). CINC-1, the rat homologue to IL-8, was added to this spectrum as a further, *68*^*th*^*cytokine,* since it had been recognized as relevant in detecting inflammatory reactions *in vitro*[24,40]. CINC-1 was measured by ELISA (Quantikine rat CINC-1, cat. no. RCN100; R&D Systems, Inc., Minneapolis, USA).

Mediator inductions were assessed as being exposure-related, if they were increased (1) in a concentration-dependent manner; and (2) by at least 2-fold as compared to the concurrent control value in the high dose group on one of the study days. Mediator levels that were below the respective detection limits were assessed as being unchanged.

Upon evaluation of this large panel of 68 antigens, MCP-1, CINC-1, M-CSF, and OPN were determined as being most relevant for characterizing pulmonary inflammation and therefore were selected for all subsequent BALF evaluations. This restricted monitoring panel was also arranged with the aim of covering different functional groups of antigens, i.e. CC chemokines (MCP-1); CXC chemokines (IL-8/CINC-1); hematopoietic cytokines (M-CSF); and inducers of proliferation of sessile cells (OPN) [1].

These four chemokines and cytokines were measured in-house using a sunrise MTP reader (Tecan AG, Switzerland) with the Magellan Software provided by the instrument producer, and making use of the following ELISA test kits:

● MCP-1 (rat MCP-1; cat. no BMS631INST; Bender MedSystems, Vienna, Austria)

● CINC-1 (Quantikine rat CINC-1, cat. no. RCN100; R&D Systems Inc., Minneapolis, USA;);

● M-CSF (Quantikine mouse M-CSF, cat no. MMC00; R&D Systems, Inc., Minneapolis, USA);

● OPN (Quantikine mouse OPN, cat. no. MOST00R&D Systems, Inc., Minneapolis, USA).

In order to evaluate if antigens were washed out of pulmonary cells into the BALF, antigens were also measured directly in **tissue homogenates of the lavaged lungs**: After bronchoalveolar lavage, the right lung portion of each animal was resected and stored at −80°C until lung tissue homogenate preparation: 0.2 g of the main lobe (lobus caudalis dexter) was mixed with 0.8 ml ice-cold Tissue Protein Extraction Reagent (T-PER, cat. no. 78510, Pierce Biotechnology, Rockford, IL, USA), supplemented with Complete Protease Inhibitor Cocktail (cat no., 11 873 580 001, Roche, Basel, Switzerland), and homogenized for up to 40 s with an Ultra-Turrax (IKA, Staufen, Germany). Finally, the resulting homogenate was centrifuged at 14,000 g and 4°C for 5 min.

Also for the mediators in the lung tissue homogenates, initial evaluations performed with lung tissues from the nano-CeO_2_ and nano-ZrO_2_ test groups aimed at determining relevant parameters, and the broad panel of 68 mediators (see above) was investigated in the samples from these test groups. The resulting comparison of mediator levels in the BALF and the lung tissue homogenates revealed that for two mediators (i.e. IL-1α and TNF-α), increases were consistently more pronounced in the lung tissue than in the BALF. Therefore, these two cytokines indicating local inflammation were included in the subsequent STIS (investigations of all SiO_2_ nanomaterials, BaSO_4_ and Al-doped nano-CeO_2_). The following test kits were used:

● IL-1α (FlowCytomix rat IL-1α Simplex Kit; cat. no. BMS8627FF; Bender MedSystems, Vienna, Austria), measured with a FACS Calibur flow cytometer (Becton Dickinson, Heidelberg, Germany) using the instrument producer’s FlowCytomix Pro Software, vs. 2.3.

● TNF-α (Quantikine rat TNF-α/TNFSF1A; cat. no. RTA100R&D Systems Inc., Minneapolis, USA), measured with a Sunrise MTP Reader (Tecan AG, Switzerland), using the instrument producer’s Magellan Software.

### Blood analysis

Blood samples from five fasted rats per dose group were collected in the mornings after overnight food withdrawal by retro-orbital venous plexus puncture under isoflurane anesthesia (Isoba®, Essex GmbH; Munich, Germany). The following **hematology parameters** were determined in the EDTA-stabilized blood with a hematology analyzer (Advia 120, Siemens Diagnostics; Fernwald, Germany): red blood cell counts, hemoglobin, hematocrit, mean corpuscular volume (MCV), mean corpuscular hemoglobin content (MCH), mean corpuscular hemoglobin concentration (MCHC), platelet counts, total white blood cell and differential blood cell counts.

In addition, **acute phase proteins** were determined in the serum:

● **Haptoglobin** (photometric assay based on the preservation of the hemoglobin peroxidase activity (Tridelta Ltd, Maynooth, Ireland)) was measured using the COBAS Fara instrument (Roche, Basel, Switzerland);

● **C-reactive protein** (ELISA; Becton Dickinson Biosciences, Heidelberg, Germany);

● α_**2**_**-macroglobulin** (ELISA; Kamiya Biomedical Company, Seattle, USA). The latter two were measured with a Sunrise MTP Reader (Tecan AG, Switzerland) using the Magellan Software provided by the instrument producer.

In animals exposed to micron-scale ZnO or coated nano ZnO, **cell mediators** were also measured in serum to further assess possible systemic effects. For this purpose, samples were sent to Rules Based Medicine, Inc. (Austin, TX, USA), and the same panel of 67 antigens was measured with xMAP technology (Luminex Corporation, Austin, TX, USA), as described for the BALF samples.

### Necropsy and histopathological evaluation

At necropsy, animals were exsanguinated by section of the *aorta abdominalis* and *vena cava* under Narcoren® anesthesia. In accordance with the provisions of OECD TG 412, the absolute and relative organ weights of the adrenal glands, brain with olfactory bulb, epididymis, heart, kidneys, liver, lungs, spleen, testes, thymus and thyroid glands were assessed. For fixation, the lungs were instilled (30 cm water column) with neutral buffered 10% formalin (corresponding to 4% formaldehyde). Likewise, the nasal cavities (4 levels) and larynxes (3 levels) were fixed in neutral buffered 10% formalin.

The organs and tissues were trimmed and sectioned according to the trimming guides for inhalation studies [68-70]. Paraffin sections were stained with hematoxylin and eosin and examined by light microscopy. Full histopathological examinations were performed in the animals of the control and high concentration groups. If changes were observed in the high concentration, groups the respective organs and tissues of the animals exposed to the low and intermediate concentrations were also examined.

### Assessment of cell proliferation and apoptosis rates

In animals exposed to ZnO, coated nano-ZnO, nano-CeO_2_, and nano-ZrO_2_, cell proliferation and apoptosis rates were determined in large/medium bronchi, terminal bronchioli, and alveoli. Three days prior to necropsy, rats were subcutaneously implanted with Alzet osmotic pumps (Model 2ML1, Alzet Corporation; Palo Alto, CA, USA) containing 20 mg/ml 5-bromo-2-deoxyuridine (BrdU). Making use of histopathological sections that were dewaxed to remove paraffin, cell proliferation was determined after immunostaining with mouse anti-BrdU antibody (BioGene; Hamburg, Germany). Labeling indices (i.e. percentage of nuclei counted undergoing replicative DNA synthesis indicating cells present in the S-phase) were determined for three pulmonary compartments, namely the large/medium sized bronchi, the terminal bronchioli and the alveoli. In each compartment, a minimum of 1,000 cells was evaluated. Apoptosis in lung sections was determined with a TUNEL kit (Roche Diagnostics; Germany) employing the manufacturers’ instructions. Counts of apoptotic cells were performed in the three pulmonary compartments mentioned above.

### Organ burden analysis and TEM and EDX organ analysis

At necropsy, the lungs, the mediastinal lymph nodes, livers, kidneys, spleens as well as the basal brains with olfactory bulbs were excised. For organ burden analysis, the organs and tissues were digested, and their test material content was analysed either by Inductively Coupled Plasma-Atomic Emission Spectrometry (ICP-AES, Varian Vista Pro; Palo Alto, CA, USA) or by Inductively Coupled Plasma-Mass Spectrometry (ICP-MS, Agilent 7500C; Agilent Technologies; Santa Clara, CA, USA). Sample solutions were nebulized with a Meinhard Nebulizer (Meinhard; CO, USA), and the respective main element of the given test substance (Ti, Zr, Si, Ba, or Ce) was analyzed at a plasma power of 1200 W (ICP-AES) or 1150 W (ICP-MS).

In addition to the analytically determined lung burden, the fraction of potential pulmonary deposition (Table 4) was calculated using the MPPD software, version 2.11 [30,31]. The MPPD model is based on the empirically determined mass median aerodynamic diameter (MMAD) and the geometric standard deviation (GSD) obtained during nose-only inhalation studies. Since the MPPD model requires indicating particle density, for all solid substances, agglomerate density, determined by Hg porosimetry, was applied. For all liquid substances, the material’s density was included, instead. Since these materials only form small agglomerates, agglomerate density approaches their physical density.

For organ analysis by TEM and EDX analysis, animals were anesthetized with isoflurane, and perfused transcardially with cacodylate buffer (resulting in euthanasia by exsanguination) and thereafter fixed with 5% glutaraldehyde. Lungs, mediastinal lymph nodes, brain and spleen were embedded in epoxy resin. Semi-thin and ultra-thin sections of the spleen were cut from 3 control animals and 3 animals exposed to SiO_2_.acrylate. Sections were assessed by TEM. For one animal, each, of the control and dose groups, EDX analysis was performed to detect silicon particles on the ultra-thin section.

### Statistical analysis

Substance-induced effects on body weight were assessed using Dunnett’s test [71,72] by comparing each dose group with the corresponding control group. All BALF data and the serum mediator data were subjected to non-parametric one-way analysis using the Kruskal-Wallis test (two-sided). If the resulting p value was 0.05 or below, a pairwise comparison of each dose group with the corresponding control group was performed using the two-sided Wilcoxon test or the two-sided Mann–Whitney U-test [73]. Cell proliferation and apoptosis data were analysed by pairwise comparison of each dose group with the corresponding control group using the one-sided Wilcoxon test.

## Abbreviations

AAN: Average agglomeration number; ALP: Alkaline phosphatase; AUC: Analytical ultracentrifugation; BALF: Bronchoalveolar lavage fluid; BET: Method of Brunauer-Emmett-Teller; CINC: Cytokine-induced neutrophil chemoattractant; CRP: C-reactive protein; D50: Medium value of the particle size distribution; DMEM/FCS: Dulbecco’s modified eagle medium supplemented with 10% fetal calf serum; EDX: Energy dispersed x-ray spectroscopy; EG: Exposure groups, i.e. groups of rats euthanized within 1–3 days after the final exposure; EGF: Epidermal growth factor; ESR+CPH: Electron spin resonance making use of centrophenoxine spin traps; ESR+DMPO: Electron spin resonance making use of dimethyl-pyrroline-N-oxide spin traps; GCP: Granulocyte chemotactic peptide; GGT: γ-Glutamyltransferase; ICP-AES: Inductively coupled plasma atomic emission spectrometry; ICP-MS: Inductively coupled plasma optical emission spectrometry; IFCC: International federation of clinical chemistry; IFN: Interferon; IP-10: Interferon inducible protein-10; IL: Interleukin; KC/GROα: Keratinocyte cytokine/Growth-regulated oncogen-α; LDH: Lactate dehydrogenase; LOQ: Limit of quantification; M-CSF: Macrophage colony stimulating factor; MCP: Monocyte chemoattractant protein; MDC: Macrophage-derived chemoattractant; MIP: Macrophage inflammatory protein; MMAD: Mass median aerodynamic diameter; MMP: Matrix metalloproteinase; MPO: Myeloperoxidase; MPPD: Multiple-path particle dosimetry model; MWCNTs: Multi-walled carbon nanotubes; NAG: N-Acetyl-β-glucosaminidase; NGAL: Neutrophil gelatinase associated lipocalin; NO: Nitric oxide; NOAEC: No observed adverse effect concentration; NOEC: No observed effect concentration; OPN: Osteopontin; PEG: Polyethyleneglycol; PMN: Polymorphonuclear; PPS: Primary particle size; RG: Recovery groups: i.e. groups of animals euthanized after the 14- or 21-day post-exposure recovery period; ROS: Reactive oxygen species; SEM: Scanning electron microscopy; SGD: Geometric standard deviation; SGOT: Serum glutamic oxaloacetic transaminase; SI: Supplementary information; SIMS: Secondary ion mass spectrometry; SMPS: Scanning mobility particle sizer; SWCNT: Single-walled carbon nanotubes; STIS: Short-term inhalation study protocol; TEM: Transmission electron microscopy; TIMP: Tissue inhibitor of metalloproteinases; TGF: Transforming growth factor; TNF: Tumor necrosis factor; TODA: Trioxadecanoic acid; VCAM: Vascular cellular adhesion molecule; VEGF: Vascular endothelial growth factor; vWF: Van Willebrand factor; XPS: X-ray photoelectron spectroscopy; XRD: X-ray diffusion.

## Competing interests

RL, LM, TH, VS, ST, WW, SG, KW, BR are employees of BASF SE, a chemical company producing and marketing nanomaterials. The TiO_2_ and the ZnO test materials are or were commercialized by BASF as cosmetic sunscreens. The non-surface functionalized SiO_2_ nanomaterial is being used by BASF for various applications. Some of the other nanomaterials are or were evaluated during product research and development.

## Authors’ contributions

RL led the projects on inhalation toxicity of nanomaterials, and designed, monitored and assessed the studies. LM carried out the inhalation experiments. VS performed the analysis of BALF. ST, KK, and SG carried out the histopathological evaluation. WW performed the physico-chemical characterization of the materials. RL, LM, MW and TH wrote the manuscript. KW and BR advised the design and the interpretation of the results. All authors read and approved the final manuscript.

## Supplementary Material

Additional file 1: Tables S1-S9 Substance-induced effects in the bronchoalveolar fluid (BALF) or lung tissue homogenates. Click here for file

Additional file 2: Tables S10-S15 Summary of incidences and severities of histopathological observations in rats exposed to TiO_2_, ZnO and CeO_2_ test materials. Click here for file
