# Supplementary material for: Application of short-term inhalation studies to assess the inhalation toxicity of nanomaterials
Source: Part Fibre Toxicol. 2014 Apr 4;11:16. doi: 10.1186/1743-8977-11-16 (PMC4113196; doi:10.1186/1743-8977-11-16)
Supplement: Additional file 1: Tables S1-S9 — Substance-induced effects in the bronchoalveolar fluid (BALF) or lung tissue homogenates. [file 1743-8977-11-16-S1.docx]

**Supplementary Information to:**

**Application of STIS to Assess the Inhalation Toxicity of 13 Nanomaterials**

*Robert Landsiedel, Lan Ma-Hock, Thomas Hofmann, Martin Wiemann, Volker Strauss, Silke Treumann, Wendel Wohlleben, Sibylle Gröters, Karin Wiench, Bennard van Ravenzwaay*

**Tables S1-S9: Substance-induced effects in the bronchoalveolar fluid (BALF) or lung tissue homogenates**

**Table S1: Historical control ranges of parameters in BALF and lung tissue homogenates of male Crl:Wi(Han) rats aged 8-12 weeks**

| **Parameters in BALF** | **Unit** | **Mean** | **Minimum** | **Maximum** |
| --- | --- | --- | --- | --- |
| Total protein | mg/L | 41 | 18 | 75 |
| GGT | nkat/L | 15 | 0 | 39 |
| LDH | µkat/L | 0.30 | 0.19 | 0.50 |
| ALP | µkat/L | 0.53 | 0.23 | 0.87 |
| NAG | nkat/L | 23 | 7 | 45 |
| Total cell counts | count/µL | 44.3 | 23.8 | 75.2 |
| Absolute macrophage counts | count/µL | 43.8 | 23.7 | 73.6 |
| Absolute lymphocyte counts | count/µL | 0.2 | 0 | 0.8 |
| Absolute PMN neutrophil counts | count/µL | 0.5 | 0.1 | 1.6 |
| Absolute monocyte counts | count/µL | 0 | 0 | 0.1 |
| MCP-1 | pg/mL | 18.5 | 14.6 | 32.6 |
| CINC-1/IL-8 | pg/mL | 62.9 | 24.6 | 146.9 |
| M-CSF | pg/mL | 14.2 | 14.0 | 15.6 |
| OPN | pg/mL | 98.7 | 37.3 | 269.0 |
| **Parameters in lung tissue** |  |  |  |  |
| IL-1α | pg/mL | 1226 | 319 | 2401 |
| TNF-α | pg/mL | 35.8 | 12.7 | 68.6 |

Footnote to Table S1:

Means of 44 sampling dates (14 dates for cytokines), 5 individuals per control group, as well as the mean, minimum, and maximum values of all studies are indicated.

Abbreviations: GGT: γ-glutamyltransferase; LDH: lactate dehydrogenase; ALP: alkaline phosphatase; NAG: N-acetyl-β-D-glucosaminidase; PMN: polymorphonuclear; CINC-1/IL-8: cytokine-induced neutrophil chemoattractant-1, the rat homologue to IL-8; MCP-1: monocyte chemoattractant protein-1; M-CSF: macrophage colony stimulating factor; OPN: osteopontin; IL-1α: interleukin 1α; TNF-α: tumor necrosis factor-α.

**Table S2: Effects induced by coated nano-TiO_2_ (T-Lite SF™) in the BALF**

|  | Exposure groups | | | Recovery groups |
| --- | --- | --- | --- | --- |
| Concentration (mg/m^3^) | 0.5 | 2 | 10 | 10 |
| **Protein / Enzymes** | | | | |
| Total protein | 0.8 | 0.9 | *2.5 | **1.7 |
| GGT | 1.1 | 1.6 | **4.9 | 2.7 |
| LDH | 1.2 | *1.4 | **4.3 | *1.7 |
| ALP | 0.9 | **1.9 | **2.5 | **2.3 |
| NAG | 1 | 1.2 | **1.5 | 1 |
| **Cell counts** | | | | |
| Total cells | 1.2 | 1.2 | **5.6 | 1.3 |
| Macrophages | 1.3 | 0.9 | 0.8 | 0.8 |
| Lymphocytes | 0.5 | 3.5 | 20.5 | + |
| PMN neutrophils | 0.4 | **7.1 | **95.2 | **25.7 |
| Monocytes | 2 | *17.0 | **448.0 | 4 |

Footnote to Table S2:

Rat inhalation exposure to coated nano-TiO_2_ (T-Lite SF^TM^) in the STIS: BALF parameter changes (mean values expressed as x-fold of concurrent mean control value) in the exposure groups 3 days after final exposure and in the recovery groups 24 days after final exposure. Cell mediators were not assessed.

Statistical evaluation (Mann-Whitney-U-test): * : p ≤ 0.05; ** : p ≤ 0.01.

+ : Value increased, but no ratio calculable, because corresponding control value = 0.

Abbreviations: GGT: γ-glutamyltransferase; LDH: lactate dehydrogenase; ALP: alkaline phosphatase; NAG: N-acetyl-β-D-glucosaminidase; PMN: polymorphonuclear; STIS: short-term inhalation study.

**Table S3: Effects induced by coated nano-ZnO or micron-scale ZnO (ZnO) in the BALF**

|  | Exposure groups | | | | Recovery groups | | | |
| --- | --- | --- | --- | --- | --- | --- | --- | --- |
|  | Coated nano-ZnO | | | ZnO | Coated nano-ZnO | | | ZnO |
| Concentration (mg/m^3^) | 0.5 | 2.5 | 12.5 | 12.5 | 0.5 | 2.5 | 12.5 | 12.5 |
| **Protein/Enzymes** |  |  |  |  |  |  |  |  |
| Total protein | 1.2 | 2.4** | 7.5** | 9.2** | 0.9 | 0.9 | 0.7 | 0.7 |
| GGT | 10.0* | 27.0 | 64.0** | 33.0** | 0.7 | 0.7 | 0.3 | 1.0 |
| LDH | 1.1 | 3.8** | 10.2** | 9.7** | 1.1 | 1.1 | 0.8 | 1.0 |
| ALP | 1.0 | 7. 2** | 22.4** | 3.7** | 0.8 | 1.0 | 0.6* | 0.6* |
| NAG | 1.0 | 2.2** | 3.6** | 4.3** | 1.0 | 1.1 | 0.9 | 1.1 |
| **Cell counts** |  |  |  |  |  |  |  |  |
| Total cells | 1.5 | 3.2** | 5.2** | 4.6** | 1.1 | 1.2 | 1.2 | 1.2 |
| Macrophages | 1.5 | 2.5* | 0.8 | 2.8** | 1.1 | 1.2 | 1.2 | 1.3 |
| Lymphocytes | 1.9 | 16.4** | 90.4** | 77.1** | 1.5 | 2.1 | 2.8 | 0.4 |
| PMN neutrophils | 2.2 | 105.5** | 649.9** | 178.3** | 0.9 | 0.6 | 0.5 | 0.1 |
| **Mediators** |  |  |  |  |  |  |  |  |
| β_2_-Microglobulin | 1.0 | 2.7** | 4.5** | 4.3** | 1.0 | 1.0 | 1.0 | 1.0 |
| CINC-1/IL-8 | 1.3* | 5.5** | 25.8** | 9.8** | 1.4 | 1.0 | 0.9 | 0.9 |
| Clusterin | 1.8* | 3.3** | 11.3** | 16.4** | 1.0 | 1.1 | 0.7 | 1.0 |
| CRP | 1.7 | 2.7** | 7.9** | 21.2** | 0.7 | 1.0 | 0.8 | 0.5 |
| Cystatin C | 0.7 | 3.1** | 14.6** | 9.5** | 1.0 | 0.8 | 0.6 | 0.5** |
| Eotaxin | 0.9 | 1.5* | 3.0** | 1.8** | 1.1 | 1.0 | 0.9 | 0.9 |
| Fibrinogen | 1.0 | 1.8** | 4.8** | 5.1** | 1.0 | 1.1 | 1.0 | 1.0 |
| GCP-2 | 1.2 | 6.7** | 16.3** | 5.5** | 1.0 | 0.9 | 0.9 | 0.9 |
| Haptoglobin | 1.0 | 1.7* | 4.7** | 4.6** | 0.8 | 0.9 | 0.8 | 0.7 |
| IL-11 | 1.0 | 1.0 | 1.1 | 2.2* | 1.2 | 1.6 | 1.4 | 1.0 |
| MCP-1 | 1.2 | 2.0** | 16.0** | 83.6** | 1.0 | 0.9 | 0.8* | 0.9 |
| MCP-3 | 1.0 | 1.3* | 6.1** | 73.0** | 1.0 | 0.8* | 0.8 | 0.9 |
| M-CSF | 0.9 | 8.4** | 23.0** | 9.8** | 1.0 | 1.0 | 0.7* | 0.8 |
| MDC | 0.9 | 9.6* | 61.0** | 77.6** | 1.1 | 0.8* | 1.0 | 1.0 |
| MIP-1α | 1.0 | 1.0** | 1.4* | 5.8** | 1.0 | 1.0 | 1.0 | 1.0 |
| MIP-2 | 1.0 | 2.2** | 4.6** | 3.0** | 1.1 | 1.0 | 1.0 | 1.0 |
| MMP-9 | 1.4 | 2.5** | 3.0** | 1.5 | 1.2 | 0.9 | 0.9 | 1.1 |
| MPO | 0.8 | 62.3** | 55.3** | 46.8** | 0.5 | 0.3 | 0.4 | 0.3 |
| Oncostatin M | 1.1 | 1.1 | 1.3* | 2.5** | 1.0 | 1.0 | 0.9 | 1.1 |
| OPN | 1.4 | 5.5** | 10.8** | 6.7** | 0.4* | 0.3* | 0.3* | 0.4 |
| Serum amyloid P | 1.4 | 1.8** | 3.2** | 3.5** | 0.9 | 1.1 | 1.0 | 0.6 |
| Stem cell factor | 1.1 | 1.1 | 1.4** | 2.6** | 1.1 | 1.0 | 0.9 | 1.0 |
| TIMP-1 | 2.1* | 1.4* | 3.3** | 1.6 | 0.6 | 0.6 | 0.8 | 0.7 |
| Thrombopoetin | 0.7 | 1.5** | 2.9** | 2.0** | 1.7 | 1.5 | 1.7 | 1.7 |
| VCAM-1 | 1.0 | 1.8** | 6.4** | 6.4** | 0.9 | 0.8 | 0.8 | 1.1 |
| VEGF | 1.1 | 3.7** | 8.8** | 6.6** | 1.0 | 0.9 | 0.7 | 0.7* |
| vWF | 1.1 | 1.6** | 3.2** | 3.7** | 1.1 | 1.0 | 1.0 | 1.1 |

Footnote to Table S3:

Rat inhalation exposure to coated nano-ZnO or micron-scale ZnO (ZnO): Parameter changes in the BALF (mean values expressed as x-fold of concurrent mean control value) in the exposure groups 3 days after final exposure and in the recovery groups 24 days after final exposure. A total of 68 cell mediators were assessed. Results are only presented for mediators, for which significant changes were recorded in at least one dose group.

Statistical evaluation (Mann-Whitney-U-test): * : p ≤ 0.05; ** : p ≤ 0.01.

Abbreviations: STIS: short-term inhalation study. ALP: alkaline phosphatase; CINC-1: cytokine-induced neutrophil chemoattractant-1; CRP: C-reactive protein; GCP: granulocyte chemotactic peptide; GGT: γ-glutamyltransferase; IL: interleukin; LDH: lactate dehydrogenase; M-CSF: macrophage colony stimulating factor; MCP: monocyte chemoattractant protein; MDC: macrophage-derived chemoattractant; MIP: macrophage inflammatory protein; MMP: matrix metalloproteinase; MPO: Myeloperoxidase; NAG: N-acetyl-β-D-glucosaminidase; OPN: osteopontin; PMN: polymorphonuclear; TIMP: tissue inhibitor of metalloproteinases; TNF-α: tumor necrosis factor-α; VCAM: vascular cellular adhesion molecule; VEGF: vascular endothelial growth factor; vWF: van Willebrand factor.

**Table S4: Clinical pathology effects induced by SiO_2_.naked, SiO_2_.acrylate, SiO_2_.PEG, SiO_2_.amino, and SiO_2_.phosphate in the BALF ^a^**

|  | **SiO_2_.naked** | | | | **SiO_2_.acrylate** | | | **SiO_2_.PEG** | **SiO_2_.amino** | **SiO_2_.phosphate** |
| --- | --- | --- | --- | --- | --- | --- | --- | --- | --- | --- |
| **Concentration (mg/m^3^)** | **0.5** | **2** | **10** | **50** | **0.5** | **2** | **10** | **50** | **50** | **50** |
| **Protein / Enzymes** | | | | | | | | | | |
| **Total Protein (EG)** | 0.9 | 1.1 | 1.0 | 1.0 | 0.6 | 0.5 | 0.6 | 1.1 | 0.7 | 1.1 |
| **(RG)** | 1.1 | 0.8 | 1.1 | 1.2 | 0.8 | 0.8 | 0.7 | 0.8 | 0.9 | 1.2 |
| **GGT (EG)** | 1.2 | 1.0 | 1.1 | 1.5 | 0.7 | 1.0 | 0.8 | 1.0 | 0.9 | 1.4 |
| **(RG)** | 0.7 | 0.8 | 0.4 | 1.0 | 0 | 0 | 3.5 | 0.9 | 0.8 | 1.1 |
| **LDH (EG)** | 0.8 | 1.0 | 1.0 | 1.3 | 1.1 | 0.8 | 0.8 | 1.4 | 1.0 | 1.3 |
| **(RG)** | 1.0 | 0.9 | 0.9 | 1.6 | 0.8 | 0.7 | 1.0 | 1.0 | 0.9 | 1.1 |
| **ALP (EG)** | 0.8 | 0.8 | 0.9 | 1.2 | 0.9 | 0.8 | 0.8 | 0.9 | 0.6 | 1.3 |
| **(RG)** | 0.6** | 0.6* | 0.5** | 1.2 | 0.9 | 1.0 | 0.8 | 0.9 | 0.8 | 1.2 |
| **NAG (EG)** | 1.2 | 0.8 | 1.1 | 1.1 | 0.8 | 1.0 | 0.9 | 1.0 | 0.9 | 1.0 |
| **(RG)** | 1.0 | 0.9 | 1.7* | 1.1 | 1.2 | 0.9 | 1.0 | 1.1 | 1.0 | 1.2 |
| **BALF cell counts** | | | | | | | | | | |
| **Total cells (EG)** | 1.0 | 1.2 | 0.9 | 1.2 | 1.1 | 1.2 | 0.9 | 0.8 | 0.8 | 1.3 |
| **(RG)** | 0.8 | 0.9 | 0.9 | 1.0 | 0.9 | 0.9 | 1.1 | 1.1 | 0.9 | 1.0 |
| **Macrophages (EG)** | 0.9 | 1.1 | 0.9 | 1.1 | 1.1 | 1.2 | 1.0 | 0.8 | 0.8 | 1.3 |
| **(RG)** | 0.9 | 0.9 | 0.9 | 0.7 | 1.0 | 0.9 | 1.1 | 1.1 | 0.8 | 1.0 |
| **Lymphocytes (EG)** | + | + | + | 5.6** | 0.2 | 0.8 | 0.2 | 1.8 | 1.1 | 3.0 |
| **(RG)** | + | + | + | 4.8* | 0.3 | 0.5 | 0.2 | 0.7 | 2.4 | 0.6 |
| **PMN neutrophils (EG)** | 0.9 | 0.8 | 0.8 | 11.2** | 0.2 | 0.3 | 0 | 2.3 | 4.2 | 2.0 |
| **(RG)** | 1.9 | 3.3 | 32.5** | 21.8** | 0.5 | 1.3 | 0.2 | 1.0 | 1.8 | 0.3 |
| **Monocytes (EG)** | + | + | + | - | + | + | + | 0.7 | - | - |
| **(RG)** | + | + | + | 2.1 | + | + | + | - | + | - |
| **Mediators in BALF** | | | | | | | | | | |
| **MCP-1 (EG)** | - | - | - | 0.9 | 0.7 | 0.9 | 1 | 1.0 | 1.0 | 1.0 |
| **(RG)** | - | - | - | 1.8 | 0.7 | 0.9 | 0.9 | 0.9 | 1.2 | 1.0 |
| **CINC-1/IL-8 (EG)** | 0.7 | 0.8 | 1.1 | 1.2 | - | - | - | 1.2 | 0.8 | 1.1 |
| **(RG)** | 1.2 | 1.1 | 1.5 | 1.5 | - | - | - | 1.0 | 0.9 | 1.2 |
| **M-CSF (EG)** | 1.0 | 1.0 | 1.0 | 1.0 | - | - | 1.0 | 1.0 | 1.0 | 1.0 |
| **(RG)** | 1.0 | 1.0 | 1.0 | 1.0 | - | - | - | 1.0 | 1.0 | 1.0 |
| **OPN (EG)** | 0.9 | 1.0 | 1.0 | 0.8 | - | - | - | 0.5 | 1.1 | 0.9 |
| **(RG)** | 1.0 | 1.3 | 2.1** | 1.8 | - | - | - | 0.4 | 1.6 | 0.8 |

Footnote to Table S4:

Rat inhalation exposure to SiO_2_ nanomaterials in the STIS: Parameter changes in the BALF (mean values expressed as x-fold of concurrent mean control value) in the exposure groups 3 days after final exposure and in the recovery groups 24 days after final exposure. Statistical evaluation (Mann-Whitney-U-test): * : p ≤ 0.05; ** : p ≤ 0.01.

+ : Value increased, but no ratio calculable, because corresponding control value = 0.

a: No data recorded for IL-1α or TNF-α in lung tissue homogenates. There were no significant findings in any of the dose groups treated with any of the SiO_2_ materials.

Abbreviations: STIS: short-term inhalation study. EG: exposure groups, i.e. groups of rats euthanized within 1-3 days after the final exposure; GGT: γ-glutamyltransferase; LDH: lactate dehydrogenase; ALP: alkaline phosphatase; NAG: N-acetyl-β-D-glucosaminidase MCP-1: monocyte chemoattractant protein-1; IL: interleukin; CINC-1: cytokine-induced neutrophil chemoattractant-1; M-CSF: macrophage colony stimulating factor; OPN: osteopontin; PMN: polymorphonuclear; RG: recovery groups: i.e. groups of animals euthanized after the 14- or 21-day post-exposure recovery period; TNF-α: tumor necrosis factor-α.

**Table S5: Clinical pathology effects induced by the BaSO_4_ and ZrO_2_ nanomaterials in the BALF and homogenated lung tissue**

|  | |  | |  | | |  | |  | |  | |  | | |  |  |  |  |
| --- | --- | --- | --- | --- | --- | --- | --- | --- | --- | --- | --- | --- | --- | --- | --- | --- | --- | --- | --- |
|  | **BaSO_4_ (NM-220)** | | | | | **nano-ZrO_2_** | | | | | | **ZrO_2_.TODA** | | | | | **ZrO_2_.acrylate** | | |
| **Concentration (mg/m^3^)** | **2** | | **10** | | **50** | **0.5** | | **2.5** | | **10** | | **2** | | **10** | **50** | | **2** | **10** | **50** |
| **Protein/Enzymes** | | | | | | | | | | | | | | | | | | | |
| **Total Protein (EG)** | 1.2 | | 1.4 | | 0.9 | 0.7 | | 0.9 | | 1.2 | | 1.1 | | 0.8 | 0.8 | | 1.4 | 1.4 | 0.8 |
| **(RG)** | 0.8 | | 0.8 | | 1.6 | 0.9 | | 1.3 | | 1.0 | | 1.1 | | 1.3 | 0.8 | | 1.0 | 0.9 | 1.3 |
| **GGT (EG)** | 1.6 | | 1.1 | | 0.9 | 0.9 | | 1.4 | | 1.6 | | 1.1 | | 1.2 | 1.9 | | 0.9 | 1.0 | 1.1 |
| **(RG)** | 1.9 | | 1.7 | | 1.2 | 0.4 | | 1.1 | | 0 | | 0.8 | | 0.1 | - | | 1.0 | 0.9 | 0.9 |
| **LDH (EG)** | 1.0 | | 1.0 | | 1.1 | 0.9 | | 0.9 | | 1.4 | | 0.9 | | 0.9 | 1.1 | | 1.0 | 1.4 | 0.6 |
| **(RG)** | 1.1 | | 1.5 | | 1.6 | 1.2 | | 1.2 | | 1.0 | | 0.9 | | 1.1 | 0.7 | | 1.0 | 1.1 | 1.0 |
| **ALP (EG)** | 0.9 | | 0.7 | | 0.8 | 1.2 | | 0.8 | | 2.3* | | 1.0 | | 0.7 | 1.0 | | 1.2 | 1.3 | 0.8 |
| **(RG)** | 1.6 | | 1.3 | | 1.0 | 1.3 | | 1.8** | | 1.4 | | 0.9 | | 1.0 | 0.8 | | 1.0 | 1.0 | 1.0 |
| **NAG (EG)** | 1.0 | | 0.9 | | 1.0 | 0.6 | | 0.7 | | 1.2 | | 1.0 | | 0.9 | 1.0 | | 1.1 | 1.3 | 0.7 |
| **(RG)** | 1.4 | | 1.2 | | 1.1 | 0.8 | | 1.4 | | 1.2 | | 1.0 | | 0.8 | 0.7 | | 0.8 | 0.8 | 1.2 |
| **BALF cell counts** | | | | | | | | | | | | | | | | | | | |
| **Total cells (EG)** | 1 | | 1.1 | | 1.1 | 0.8 | | 0.9 | | 0.9 | | 1.0 | | 1.1 | 0.8 | | 0.9 | 1.0 | 1.0 |
| **(RG)** | 0.9 | | 1 | | 0.8 | 1 | | 1 | | 1.1 | | 0.9 | | 0.6 | 0.6 | | 1.0 | 1.1 | 0.9 |
| **Macrophages (EG)** | 0.9 | | 1.1 | | 1 | 0.8 | | 0.9 | | 0.9 | | 1.0 | | 1.1 | 0.7 | | 0.8 | 0.9 | 1.1 |
| **(RG)** | 0.8 | | 1 | | 0.8 | 1 | | 0.9 | | 1.1 | | 0.9 | | 0.6 | 0.7 | | 0.9 | 1.0 | 0.9 |
| **Lymphocytes (EG)** | 2.7 | | 2.7 | | 1 | + | | + | | + | | 0.7 | | 1.5 | 1.4 | | 1.2 | 1.5 | 2.6 |
| **(RG)** | + | | +** | | +* | + | | + | | + | | 1.1 | | 1.3 | 0.5 | | 0.8 | 0.9 | 0.7 |
| **PMN neutrophils (EG)** | 4.8 | | 1.7 | | 2.7 | 1.2 | | 1.5 | | 2.1 | | 1.6 | | 1.7 | *8.0 | | 1.7 | 0.8 | 1.6 |
| **(RG)** | 2.9 | | 2.2 | | 4.1 | 2.3 | | 2 | | 2 | | 9.1 | | 5.2 | 0.8 | | 1.5 | 0.9 | 2.3 |
| **Monocytes (EG)** | + | | + | | + | + | | + | | + | | + | | + | + | | + | + | + |
| **(RG)** | + | | + | | + | + | | + | | + | | + | | - | - | | - | + | - |
| **Mediators in the BALF** | | | | | | | | | | | | | | | | | | | |
| **MCP-1 (EG)** | 1.2 | | 1.2 | | 1.3 | - | | - | | - | | - | | - | - | | 1.3 | 1.0 | 1.1 |
| **(RG)** | 0.7 | | 0.9 | | 1.6 | - | | - | | - | | 1.0 | | 1.0 | 1.1 | | 1.0 | 4.3 | 0.2 |
| **CINC-1/IL-8 (EG)** | 1.0 | | 1.1 | | 1.2 | - | | - | | - | | 1.0 | | 1.4 | 1.2 | | 1.6 | 1.7 | 0.4 |
| **(RG)** | 1.0 | | 1.2 | | 1.4 | - | | - | | - | | 1.2 | | 1.5 | 0.9 | | 0.9 | 0.6 | 1.4 |
| **M-CSF (EG)** | 1.0 | | 1.0 | | 1.0 | - | | - | | - | | 1.0 | | 1.0 | 1.0 | | 1.0 | 1.0 | 1.0 |
| **(RG)** | 1.0 | | 1.0 | | 1.0 | - | | - | | - | | 1.0 | | 1.0 | 1.0 | | 1.0 | 1.4 | 0.7 |
| **OPN (EG)** | 0.8 | | 1.4 | | 1.5 | - | | - | | - | | 1.9 | | 1.6 | 0.8 | | 0.9 | 1.0 | 0.9 |
| **(RG)** | 0.4 | | 0.9 | | 1.3 | - | | - | | - | | 0.7 | | 0.4 | 0.8 | | 2.1 | 1.5 | 0.8 |
|  | 1.1 | | 1.2 | | 1.6* |  | |  | |  | |  | |  |  | |  |  |  |
| **Mediators in the lung tissue** | | | | | | | | | | | | | | | | | | | |
| **IL 1-α (EG)** | 1.1 | | 1.2 | | 1.6* | - | | - | | - | | - | | - | - | | - | - | - |
| **(RG)** | 1.2 | | 1.3 | | 1.1 | - | | - | | - | | - | | - | - | | - | - | - |
| **TNF-α (EG)** | 1.2 | | 1.2 | | 1.1 | - | | - | | - | | - | | - | - | | - | - | - |
| **(RG)** | 1.0 | | 1.0 | | 0.9 | - | | - | | - | | - | | - | - | | - | - | - |

Footnote to Table S5:

Rat inhalation exposure to BaSO_4_ and ZrO_2_ nanomaterials in the BALF or homogenated lung tissue: Parameter changes (mean values expressed as x-fold of concurrent mean control value) in the exposure groups 3 days after final exposure and in the recovery groups 24 days after final exposure.

For nano-ZrO_2_, a total of 68 antigens were assessed in the BALF and lung tissue, respectively, without any significant findings.

Statistical evaluation (Mann-Whitney-U-test): * : p ≤ 0.05; ** : p ≤ 0.01.

+ : Value increased, but no ratio calculable, because corresponding control value = 0.

Abbreviations: EG: exposure groups, i.e. groups of rats euthanized within 1-3 days after the final exposure; GGT: γ-glutamyltransferase; LDH: lactate dehydrogenase; ALP: alkaline phosphatase; NAG: N-acetyl-β-D-glucosaminidase MCP-1: monocyte chemoattractant protein-1; IL: interleukin; CINC-1: cytokine-induced neutrophil chemoattractant-1; M-CSF: macrophage colony stimulating factor; MCP: Monocyte chemoattractant protein; OPN: osteopontin; PMN: polymorphonuclear; RG: recovery groups: i.e. groups of animals euthanized after the 14- or 21-day post-exposure recovery period; TNF: tumor necrosis factor.

**Table S6: Effects induced by nano-CeO_2_ in the BALF**

|  | Exposure groups | | | Recovery groups | | |
| --- | --- | --- | --- | --- | --- | --- |
| Concentration (mg/m^3^) | 0.5 | 2.5 | 10 | 0.5 | 2.5 | 10 |
| **Protein/Enzymes** | | | | | | |
| Total protein | 0.6* | 1.4 | 2.8** | 1.1 | 1.2 | 1.4 |
| GGT | 2.1 | 6.4** | 6.6** | 4 | 4.1 | 5.4 |
| LDH | 1.1 | 2.2** | 4.3** | 1.2 | 1.8* | 2.3** |
| ALP | 1.0 | 2.9* | 4.1** | 1.7 | 2.0** | 2.4** |
| NAG | 0.4 | 1.4 | 3.3** | 1.2 | 1.6* | 1.7** |
| **Cell counts** | | | | | | |
| Total cells | 1.3 | 1.3 | 2.6 | 1.4 | 1.2 | 2.6 |
| Macrophages | 1.3 | 0.4* | 0.4** | 1.4 | 1.1 | 0.9 |
| Lymphocytes | 2.7* | 10.3** | 26.2** | 0.8 | 3.3* | 5.5* |
| PMN neutrophils | 4.6* | 164.1** | 386.6** | 0.9 | 8.0** | 46.7** |
| **Mediators** | | | | | | |
| β2-Microglobulin | 1.2 | 2.5** | 3.5** | 1.0 | 1.4 | 1.8* |
| CINC-1/IL-8 | 1.1 | 3.9* | 5.3** | 1.2 | 1.7* | 2.1** |
| Clusterin | 0.6 | 1.8 | 3.6** | 1.0 | 1.4 | 1.1 |
| Cystatin C | 0.9 | 1.8* | 3.8** | 2.7** | 3.3** | 2.6** |
| Eotaxin | 1.2 | 1.5* | 2.3** | 1.0 | 1.1* | 1.1 |
| Fibrinogen | 1.0 | 1.4 | 2.7** | 1.1 | 1.4 | 0.9 |
| GCP-2 | 0.7 | 10.8** | 15.4** | 1.4* | 2.8** | 5.8** |
| IP-10 | 1.1 | 1.6 | 2.3* | 0.9 | 0.9 | 0.7 |
| MCP-1 | 1.3 | 5.2** | 21.9** | 0.7 | 1.4** | 3.3** |
| MCP-3 | 1.3 | 6.6** | 24.8** | 1.0 | 1.3 | 1.9** |
| M-CSF | 1.3* | 3.4** | 9.5** | 1.0 | 1.2* | 1.4** |
| MDC | 1.7 | 79.0** | 362.5** | 1.1 | 1.2 | 3.3 |
| MIP-1β | 0.8 | 1.4 | 2.8** | 0.9 | 1.0 | 1.0 |
| MIP-2 | 0.8 | 2.8* | 2.9* | 1.0 | 1.0 | 0.9 |
| MPO | 1.1 | 55.5** | 115.0** | 2.9 | 6.2* | 21.3** |
| VEGF | 1.2 | 3.1** | 15.7** | 1.7* | 1.6* | 1.9* |

Footnote to Table S6:

Rat inhalation exposure to nano-CeO_2_ in the STIS: Changes in BALF parameters (mean values expressed as x-fold of concurrent mean control value) in the exposure groups 3 days after final exposure and in the recovery groups 24 days after final exposure. A total of 68 antigens was assessed. Results are only presented for mediators, for which significant changes were recorded in at least one dose group.

Statistical evaluation (Mann-Whitney-U-test): * : p ≤ 0.05; ** : p ≤ 0.01.

Abbreviations: STIS: short-term inhalation study; GGT: γ-glutamyltransferase; LDH: lactate dehydrogenase; ALP: alkaline phosphatase; NAG: N-acetyl-β-D-glucosaminidase; CINC-1: cytokine induced neutrophil chemoattractant-1; GCP-2: granulocyte chemotactic peptide-2; IP-10: interferon inducible protein-10; MCP: monocyte chemoattractant protein; M-CSF: macrophage colony stimulating factor; MDC: macrophage-derived chemoattractant; MIP: macrophage inflammatory protein; MPO: myeloperoxidase; OPN: osteopontin; PMN: polymorphonuclear; VEGF: vascular endothelial growth factor.

**Table S7: Effects induced by nano-CeO_2_ in the lung tissue homogenates**

|  | Exposure groups | | | Recovery groups | | |
| --- | --- | --- | --- | --- | --- | --- |
| Concentration (mg/m^3^) | 0.5 | 2.5 | 10 | 0.5 | 2.5 | 10 |
| **Mediators** |  |  |  |  |  |  |
| CINC-1/IL-8 | 1.2 | 3.2** | 4.0** | 1.1 | 1.2* | 1.7** |
| KC/GROα | 1.0 | 4.2** | 4.5** | 1.0 | 1.3* | 2.0** |
| MCP-1 | 0.8 | 1.8** | 2.5** | 1.1 | 1.2 | 1.3* |
| MCP-3 | 1.0 | 1.7** | 2.0** | 1.1 | 1.3* | 1.3 |
| M-CSF | 1.1 | 1.4** | 2.1** | 0.9 | 1.0 | 1.1 |
| MDC | 0.9 | 3.0** | 7.6** | 0.8 | 1.5 | 1.8* |
| MIP-1α | 1.5* | 1.7* | 2.3** | 1.2 | 1.0 | 1.4 |
| MIP-2 | 0.9 | 3.3** | 3.0** | 1.1 | 1.2 | 1.6* |
| NGAL | 1.0 | 2.0* | 2.6** | 1.0 | 1.0 | 1.4** |

Footnote to Table S7:

Rat inhalation exposure to nano-CeO_2_ in the STIS: Parameter changes (mean values expressed as x-fold of concurrent mean control value) in the exposure groups 3 days after final exposure and in the recovery groups 24 days after final exposure. A total of 68 antigens was assessed. Results are only presented for mediators, for which significant changes were recorded in at least one dose group.

Statistical evaluation (Mann-Whitney-U-test): * : p ≤ 0.05; ** : p ≤ 0.01.

Abbreviations: CINC-1: cytokine induced neutrophil chemoattractant-1; IL: interleukin; KC/GROα: keratinocyte cytokine/growth-regulated oncogen-α; MCP: monocyte chemoattractant protein; M-CSF: macrophage colony stimulating factor; MDC: macrophage-derived chemoattractant; MIP: macrophage inflammatory protein; MPO: myeloperoxidase; NGAL: neutrophil gelatinase associated lipocalin.

**Table S8: Effects induced by Al-doped nano-CeO_2_ in the BALF**

|  | Exposure groups | | | Recovery groups | | |
| --- | --- | --- | --- | --- | --- | --- |
| Concentration (mg/m^3^) | 0.5 | 2.5 | 10 | 0.5 | 2.5 | 10 |
| **Protein/Enzymes** | | | | | | |
| Total protein | 1.1 | 1.6** | 5.4** | 0.9 | 1.0 | 1.1 |
| GGT | 2.5* | 4.1** | 4.9** | 2 | 1.9* | 3.6** |
| LDH | 1.2 | 2.5** | 4.8** | 1.3 | 1.5* | 2.5** |
| ALP | 1.6* | 2.1** | 2.7** | 2 | 1.9 | 2.3 |
| NAG | 1.2 | 1.2* | 2.1** | 1.1 | 1.2 | 1.4 |
| **Cell counts** | | | | | | |
| Total cells | 1.5* | 1.1 | 3.3** | 1.0 | 1.1 | 1.0 |
| Macrophages | 1.4 | 0.8 | 0.7 | 0.9 | 1.1 | 0.8 |
| Lymphocytes | 0 | 1.7 | 2.3 | + | +* | +* |
| PMN neutrophils | 14.7* | 42.6** | 339.9** | 7.1** | 4.8* | 33.5** |
| **Mediators** |  |  |  |  |  |  |
| CINC-1/IL-8 | 1.4 | 2.6** | 7.9** | 1.3 | 1.4 | 2.1 |
| Clusterin | 1.0 | 1.0 | 1.0 | 1.0 | 1.0 | 1.0 |
| MCP-1 | 1.2 | 2.2** | 25.1** | 0.8 | 0.9 | 2.9* |
| M-CSF | 1.0 | 1.0 | 1.4 | 1.0 | 1.0 | 1.0 |
| OPN | 1.0 | 2.2 | 5.7* | 0.6 | 0.5 | 1.6 |

Footnote to Table S8

Rat inhalation exposure to Al-doped nano-CeO_2_ in the STIS: Parameter changes in the BALF (mean values expressed as x-fold of concurrent mean control value) in the exposure groups 3 days after final exposure and in the recovery groups 24 days after final exposure.

Statistical evaluation (Mann-Whitney-U-test): * : p ≤ 0.05; ** : p ≤ 0.01.

+ : Value increased, but no ratio calculable, because corresponding control value = 0.

Abbreviations: GGT: γ-glutamyltransferase; LDH: lactate dehydrogenase; ALP: alkaline phosphatase; NAG: N-acetyl-β-D-glucosaminidase; PMN: polymorphonuclear; CINC-1: cytokine induced neutrophil chemoattractant-1; IL: interleukin; MCP: monocyte chemoattractant protein; M-CSF: macrophage colony stimulating factor; OPN: osteopontin.

**Table S9: Effects induced by Al-doped nano-CeO_2_ in the lung tissue homogenate ^a^**

|  | Exposure groups | | | Recovery groups | | |
| --- | --- | --- | --- | --- | --- | --- |
| Concentration (mg/m^3^) | 0.5 | 2.5 | 10 | 0.5 | 2.5 | 10 |
| **Mediators** | | | | | | |
| IL-1α | 1.4* | 3.3** | 4.4** | 1.0 | 1.0 | 1.5* |
| TNF-α | 1.6 | 1.4 | 1.8 | 1.1 | 0.9 | 1.4 |

Footnote to Table S9

a: Rat inhalation exposure to Al-doped nano-CeO_2_ in the STIS: Changes in mediator levels in lung tissue homogenates (mean values expressed as x-fold of concurrent mean control value) in the exposure groups 3 days after final exposure and in the recovery groups 24 days after final exposure.

Statistical evaluation (Mann-Whitney-U-test): * : p ≤ 0.05; ** : p ≤ 0.01.

Abbreviations: IL-1α: interleukin 1α; TNF-α: tumor necrosis factor-α.
