# Supplementary material for: Application of short-term inhalation studies to assess the inhalation toxicity of nanomaterials
Source: Part Fibre Toxicol. 2014 Apr 4;11:16. doi: 10.1186/1743-8977-11-16 (PMC4113196; doi:10.1186/1743-8977-11-16)
Supplement: Additional file 2: Tables S10-S15 — Summary of incidences and severities of histopathological observations in rats exposed to TiO2, ZnO and CeO2 test materials. [file 1743-8977-11-16-S2.doc]

**Supplementary Information to:**

**Application of STIS to Assess the Inhalation Toxicity of 13 Nanomaterials**

*Robert Landsiedel, Lan Ma-Hock, Thomas Hofmann, Martin Wiemann, Volker Strauss, Silke Treumann, Wendel Wohlleben, Sibylle Gröters, Karin Wiench, Bennard van Ravenzwaay*

**Tables S10-S15: Summary of incidences and severities of histopathological observations in rats exposed to TiO_2_, ZnO and CeO_2_ test materials**

**Table S10: Summary of incidences and severities of main histopathologic pulmonary observations in male Wistar rats exposed to coated nano-TiO_2_ (T-Lite SF^TM^)**

|  | **Exposure group** | | | |
| --- | --- | --- | --- | --- |
| **Concentration (mg/m^3^)** | **0** | **0.5** | **2** | **10** |
| No. of animals examined | 3 |  |  | 3 |
| **Histiocytosis, diffuse** |  |  |  | **3** |
| - *Grade 1* |  |  |  | *2* |
| - *Grade 2* |  |  |  | *1* |
| **Pigment-loaded macrophages** |  |  |  | **3** |
| - *Present* |  |  |  | *3* |

**Table S11: Summary of incidences and severities of main histopathologic pulmonary observations in male Wistar rats exposed to coated nano-ZnO or micron-scale ZnO (ZnO)**

|  | **Exposure groups** | | | | | **Recovery groups** | | | | |
| --- | --- | --- | --- | --- | --- | --- | --- | --- | --- | --- |
| **Test substance** |  | **Coated nano-ZnO** | | | **ZnO** |  | **Coated nano-ZnO** | | | **ZnO** |
| **Concentration (mg/m^3^)** | **0** | **0.5** | **2.5** | **12.5** | **12.5** | **0** | **0.5** | **2.5** | **12.5** | **12.5** |
| No. of animals examined | 6 | 6 | 6 | 6 | 6 | 6 | 6 | 6 | 6 | 6 |
| **Granulocyte infiltration, (multi)focal** |  |  |  | **3** | **5** |  |  |  |  |  |
| - *Grade 1* |  |  |  | *2* | *3* |  |  |  |  |  |
| - *Grade 2* |  |  |  | *1* | *2* |  |  |  |  |  |
| **Hyperplasia, bronchio-alveolar** |  |  |  |  | **4** |  |  |  |  |  |
| - *Grade 1* |  |  |  |  | *2* |  |  |  |  |  |
| - *Grade 2* |  |  |  |  | *2* |  |  |  |  |  |
| **Histiocytosis, diffuse** |  |  |  |  | **2** |  |  |  |  |  |
| - *Grade 2* |  |  |  |  | *2* |  |  |  |  |  |
| **Histiocytosis, (multi)focal** | **4** | **5** | **6** | **6** | **3** | **2** | **6** | **6** | **6** | **6** |
| - *Grade 1* | *4* | *2* | *3* | *-* | *-* | *2* | *4* | *2* | *3* | *-* |
| - *Grade 2* | *-* | *3* | *3* | *5* | *3* | *-* | *2* | *4* | *2* | *6* |
| - *Grade 3* | *-* | *-* | *-* | *1* | *-* | *-* | *-* | *-* | *1* | *-* |

**Table S12: Summary of incidences and severities of main histopathologic observations in the nasal cavity in male Wistar rats exposed to coated nano-ZnO or micron-scale ZnO (ZnO)**

|  | **Exposure groups** | | | | | **Recovery groups** | | | | |
| --- | --- | --- | --- | --- | --- | --- | --- | --- | --- | --- |
| **Test substance** |  | **Coated nano-ZnO** | | | **ZnO** |  | **Coated nano-ZnO** | | | **ZnO** |
| **Concentration (mg/m^3^)** | **0** | **0.5** | **2.5** | **12.5** | **12.5** | **0** | **0.5** | **2.5** | **12.5** | **12.5** |
| No. of animals examined | 6 | 6 | 6 | 6 | 6 | 6 | 6 | 6 | 6 | 6 |
| **Necrosis of olfactory epithelium** |  | **1** | **4** | **6** | **6** |  |  |  |  |  |
| - *Grade 1* |  | *1* | *3* | *-* | *-* |  |  |  |  |  |
| - *Grade 2* |  | *-* | *1* | *4* | *-* |  |  |  |  |  |
| - *Grade 3* |  | *-* | *-* | *2* | *6* |  |  |  |  |  |
| **Irregular olfactory epithelium** |  |  |  |  |  |  |  |  | **1** | **3** |
| - *Grade 2* |  |  |  |  |  |  |  |  | *1* | *3* |

**Table S13: Effects of inhalation exposure to coated nano-ZnO or micron-scale ZnO (ZnO) on cell proliferation rates in the lung**

|  | **Exposure groups** | | | | | **Recovery groups** | | | | |
| --- | --- | --- | --- | --- | --- | --- | --- | --- | --- | --- |
| **Substance** | **Control** | **Coated nano-ZnO** | | | **ZnO** | **Control** | **Coated nano-ZnO** | | | **ZnO** |
| **Concentration (mg/m^3^)** | **0** | **0.5** | **2.5** | **12.5** | **12.5** | **0** | **0.5** | **2.5** | **12.5** | **12.5** |
| Large/medium bronchi | 0.88  (1.29)  *100* | 0.89  (0.74)  *101* | 0.85  (0.46)  *97* | 1.32  (1.09)  *150* | **6.33 ****  **(3.15)**  ***719*** | 1.64  (0.69)  *100* | 1.84  (0.70)  *112* | 1.15  (0.39)  *70* | 0.86  (0.40)  *52* | 1.62  (0.46)  *99* |
| Terminal bronchioli | 1.02  (0.67)  *100* | **1.86 ***  **(0.72)**  ***182*** | **2.53 ***  **(1.56)**  ***248*** | **4.96 ****  **(2.03)**  ***460*** | **11.14 ****  **(3.65)**  ***1092*** | 2.01  (0.85)  *100* | 2.41  (0.35)  *120* | 1.14  (0.26)  *57* | 0.66  (0.25)  *33* | 1.41  (0.57)  *70* |
| Alveoli | 4.74  (2.04)  *100* | **9.18 ***  **(5.35)**  ***194*** | 6.27  (1.71)  *132* | 7.39  (3.61)  *156* | **9.98 ***  **(4.68)**  ***211*** | 3.12  (0.84)  *100* | 3.38  (0.89)  *108* | 3.27  (0.57)  *105* | 3.86  (0.73)  *124* | 4.07  (1.35)  *130* |

Footnote to Table S13:

Results are presented as mean labelling indices (i.e. percentage of nuclei counted undergoing replicative DNA synthesis) for groups of 6 rats. Figures in parentheses indicate standard deviation. Italic figures indicate increases relative to corresponding control levels. Values significantly different (on-sided Wilcoxon test) from control are: * p < 0-05; ** p < 0.01.

**Table S14: Summary of incidences and severities of main histopathologic pulmonary observations in male Wistar rats exposed to nano-CeO_2_**

|  | **Exposure groups** | | | | **Recovery groups** | | | |
| --- | --- | --- | --- | --- | --- | --- | --- | --- |
| **Concentration (mg/m^3^)** | **0** | **0.5** | **2.5** | **10** | **0** | **0.5** | **2.5** | **10** |
| No. of animals examined | 6 | 6 | 6 | 6 | 6 | 6 | 6 | 6 |
| **Histiocytosis, diffuse** |  |  |  |  |  |  |  | **2** |
| - *Grade 1* |  |  |  |  |  |  |  | *2* |
| **Histiocytosis, (multi)focal** |  |  |  |  |  | **2** | **2** | **4** |
| - *Grade 1* |  |  |  |  |  | *2* | *1* | *2* |
| - *Grade 2* |  |  |  |  |  |  | *1* | *2* |
| **Particles in macrophages** |  | **6** | **6** | **6** |  | **6** | **6** | **6** |
| - *Present* |  | *6* | *6* | *6* |  | *6* | *6* | *6* |

**Table S15: Summary of incidences and severities of main histopathologic pulmonary observations in male Wistar rats exposed to Al-doped nano-CeO_2_**

|  | **Exposure groups** | | | | **Recovery groups** | | | |
| --- | --- | --- | --- | --- | --- | --- | --- | --- |
| **Concentration (mg/m^3^)** | **0** | **0.5** | **2.5** | **10** | **0** | **0.5** | **2.5** | **10** |
| No. of animals examined | 3 | 3 | 3 | 2 | 3 | 3 | 3 | 2 |
| **Macrophage aggregation** |  |  | **3** | **2** |  |  | **1** | **2** |
